# Minimal expression of dysferlin prevents development of dysferlinopathy in dysferlin exon 40a knockout mice

**DOI:** 10.1186/s40478-022-01473-x

**Published:** 2023-01-18

**Authors:** Joe Yasa, Claudia E. Reed, Adam M. Bournazos, Frances J. Evesson, Ignatius Pang, Mark E. Graham, Jesse R. Wark, Brunda Nijagal, Kim H. Kwan, Thomas Kwiatkowski, Rachel Jung, Noah Weisleder, Sandra T. Cooper, Frances A. Lemckert

**Affiliations:** 1 Kids Neuroscience Centre, The Children’s Hospital at Westmead, Cnr Hawkesbury Road, Hainsworth Street, Westmead, Sydney, NSW 2145 Australia; 2 Functional Neuromics, Children’s Medical Research Institute, Westmead, Sydney, NSW Australia; 3 Synapse Proteomics, Children’s Medical Research Institute, The University of Sydney, Westmead, NSW Australia; 4 Operations, Children’s Medical Research Institute, The University of Sydney, Westmead, NSW Australia; 5 Metabolomics Australia, Bio21 Institute, The University of Melbourne, Victoria, Australia; 6 West Chester University, West Chester, PA 19383 USA; 7 Department of Physiology and Cell Biology, Dorothy M. Davis Heart and Lung Research Institute, The Ohio State University Wexner Medical Center, Columbus, OH 43210-1252 USA; 8 Discipline of Child and Adolescent Health, Faculty of Medicine, University of Sydney, Sydney, NSW Australia

## Abstract

Dysferlin is a Ca^2+^-activated lipid binding protein implicated in muscle membrane repair. Recessive variants in *DYSF* result in dysferlinopathy, a progressive muscular dystrophy. We showed previously that calpain cleavage within a motif encoded by alternatively spliced exon 40a releases a 72 kDa C-terminal minidysferlin recruited to injured sarcolemma. Herein we use CRISPR/Cas9 gene editing to knock out murine *Dysf* exon 40a, to specifically assess its role in membrane repair and development of dysferlinopathy. We created three *Dysf* exon 40a knockout (40aKO) mouse lines that each express different levels of dysferlin protein ranging from ~ 90%, ~ 50% and ~ 10–20% levels of wild-type. Histopathological analysis of skeletal muscles from all 12-month-old 40aKO lines showed virtual absence of dystrophic features and normal membrane repair capacity for all three 40aKO lines, as compared with dysferlin-null BLAJ mice. Further, lipidomic and proteomic analyses on 18wk old quadriceps show all three 40aKO lines are spared the profound lipidomic/proteomic imbalance that characterises dysferlin-deficient BLAJ muscles. Collective results indicate that membrane repair does not depend upon calpain cleavage within exon 40a and that ~ 10–20% of WT dysferlin protein expression is sufficient to maintain the muscle lipidome, proteome and membrane repair capacity to crucially prevent development of dysferlinopathy.

**Supplementary Information:**

The online version contains supplementary material available at 10.1186/s40478-022-01473-x.

## Introduction

Autosomal recessive variants in the *DYSF* gene, encoding dysferlin, give rise to dysferlinopathy. This late-onset, progressive muscular dystrophy encompasses two main clinical presentations; dysferlin-related limb girdle muscular dystrophy R2 (primarily affects proximal muscles) and Miyoshi myopathy type 1 (primarily affects distal muscles) [1]. Disease onset usually occurs in late teens to early adulthood with affected individuals presenting with elevated serum creatine kinase (indicative of muscle damage), progressive muscle weakness and wasting that often leads to non-ambulance [2]. There is currently no curative treatment for dysferlinopathy, though many promising therapies [3–5], including gene therapy (NCT02710500), are currently being pursued.

Dysferlin is a large transmembrane protein (237 kDa) belonging to the ferlin family of vesicle fusion proteins [6]. Ferlins are characterised by the presence of multiple C2 domains, calcium-dependent lipid/protein interacting domains found in the synaptotagmins [7, 8]. Dysferlin is highly expressed in skeletal muscles at the sarcolemma, T-tubules, sarcoplasmic reticulum and intracellular vesicles, but is also present in several other tissues including brain, endothelium, heart, kidney, monocytes, pancreas and syncytiotrophoblasts [9, 10]. Studies have linked dysferlin with multiple roles including vesicular-mediated membrane repair [11], vesicle trafficking and fusion [12], T-tubulogenesis [13], regulation of excitation–contraction coupling [14] and lipid metabolism [15, 16]. Indeed, dysferlin deficient mice have defective calcium-dependent sarcolemmal repair and display a progressive muscular dystrophy that mirrors the pathology seen in biopsies from affected individuals [11, 17].

One of the striking features of dysferlinopathy in both mice and humans is the presence of lipid droplets in muscle cells and progressive replacement of muscle cells with adipocytes [17]. Studies have shown that prior to the onset of dystrophic pathology, dysferlinopathic muscles exhibit early abnormalities such as poor membrane repair capacity, sarcolemmal tears, accumulation of vesicles and vacuoles beneath the sarcolemma, and alterations in the muscle lipidome [15, 16, 18, 19]. To date there is limited understanding of factors that lead to the lipid imbalance in dysferlinopathy that may contribute to, or even primarily underlie, the membrane repair defect observed in dysferlin-deficient muscle fibers. Analysis of pre-dystrophic skeletal muscle fibers may provide insight into the crux of the pathological abnormalities that lead to progressive loss of muscle and lipid accumulation in dysferlinopathy and provide clues to potential therapeutic targets.

It is thought that dysferlin assists skeletal muscle membrane repair by responding to the calcium-influx to aggregate available lipid vesicles or t-tubule membranes to help plug the membrane tear [20]. Our group established that following membrane injury, rapid influx of extracellular calcium triggers activation of calpain-1 and/or calpain-2, which then cleave dysferlin at protein residues encoded by the alternatively spliced exon 40a [21, 22]. This releases a 72 kDa C-terminal fragment named mini-dysferlin_C72_ comprising two C2 domains, the transmembrane domain and region immediately preceding the transmembrane domain that binds with high affinity to exposed phosphotidylserine at sites of membrane injury [23]. Calpains also remodel the cytoskeleton and plasma membrane to aid movement and fusion of membrane repair vesicles [24, 25]. Loss of calpains-1 and -2, by knockout of the shared calpain small subunit 1 (*CAPNS1*), impairs calcium-dependent membrane repair in cells exposed to mechanical scrape injury, and induces severe muscular dystrophy in mice [26]. Mini-dysferlin_C72_-containing vesicles traffic to sites of membrane injury, where they associate with MG53 (TRIM72), an E3-ubiquitin ligase that is rapidly recruited at injury sites in a calcium-independent manner [21]. Mini-dysferlin_C72_-containing vesicles and MG53 via their interaction with phosphatidylserine [27], promote fusion of vesicles to each other and to the sarcolemma forming a lattice-like network that expands from the edges of the lesion to reseal the membrane injury [21]. Other models propose the involvement of endocytosis/exocytosis of endosomal and/or lysosomal vesicles and secretion of enzymes and cytokines at the wound site that promote membrane repair [20, 28, 29]. In addition, dysferlin-mediated vesicular repair is aided by a complex interaction with several other proteins that play integral roles in membrane repair including annexins [30], caveolin-3 [31], AHNAK [32] and syntaxin-4 [33].

Using a ballistics injury model on cultured human myotubes, we previously demonstrated that dysferlin could only be detected at sites of membrane injury using a C-terminal antibody (Hamlet-1) and not other antibodies that target an N-terminal epitope (e.g. Romeo-1) or middle residues of dysferlin (e.g. anti-C2DE (SAB2100636)) [21, 22]. We further showed that injury-activated cleavage of dysferlin was mediated by calpains-1 or 2 with the cleavage motif encoded by exon 40a [26]. A recent study by Ballouhey et al. [34] further refines the calpain cleavage site to include the first 11 amino acids in the peptide encoded by dysferlin exon 40a. They also showed that exon 40a peptides are resistant to point mutations and are essential in membrane repair of human myoblasts [34].

Our objective in the current study was to specifically assess the importance of the calpain cleaved form of dysferlin in membrane repair and development of dysferlinopathy in a mouse model that lacks expression of dysferlin isoforms containing exon 40a. We used CRISPR/Cas-9 gene editing to create three *Dysf* exon 40a knockout (KO) mouse lines, each carrying different genomic deletions in and around exon 40a. Due to splicing aberrations affecting *Dysf* mRNA, the three *Dysf* exon 40a knockout lines (from here on referred to as 40aKO) express variable amounts of the canonical dysferlin isoform (NP_001124450.1) ranging from ~ 90% in 40aKO-3_high_, ~ 50% in 40aKO-2_mid_ and ~ 10–20% in 40aKO-1_low_. These models provided a unique opportunity to simultaneously evaluate the effect of *Dysf* exon 40a knockout and reduced dysferlin protein expression upon the skeletal muscle lipidome, proteome, membrane repair capacity and development of dysferlinopathy.

## Results

### Generation and characterisation of Dysferlin exon40a knockout mouse lines

Three mouse lines were generated using CRISPR/Cas9 gene editing (see Additional file 7: supplementary materials and methods) in which exon 40a of dysferlin was effectively knocked out. Different genomic deletions in this region resulted in a combination of: (1) absence of exon 40a containing *Dysf* transcripts; (2) stochastic use of an alternative cryptic acceptor splice site producing a variant *Dysf* mRNA encoding a premature termination codon; and (3) varying levels of remnant *Dysf* mRNA encoding the canonical skeletal muscle dysferlin isoform that lacks exon 40a (NM_001130978.2) (Additional file 1: Fig. S1). Consequently, the three 40aKO mouse lines were variably hypomorphic for dysferlin protein (NP_001124450.1) (Additional file 2: Fig. S2) and lack protein expression of exon 40a residues and so cannot make minidysferlin_C72_ (Additional file 16: Fig S7). Line 40AKO-1_low_ expresses approximately 10–20% of the usual dysferlin protein levels of WT mice, line 40AKO-2_mid_ expresses approximately 50% of the dysferlin protein and line 40AKO-3_high_ expresses 90–100% of WT dysferlin protein (Additional file 2: Fig. S2). Immunofluorescent staining for dysferlin showed a normal pattern of dysferlin expression in all 40aKO mice, with predominant expression at the sarcolemma (Additional file 2: Fig. S2A). Studying all three lines simultaneously allows us to discriminate the consequences of knockout of exon 40a versus the consequences of reduced dysferlin protein expression. In addition, we bred the 40aKO-1_low_ line with BLAJ to further reduce dysferlin protein expression. We studied the F1 mice for development of histopathology at 12 months of age. However, we were unable to confirm dysferlin protein levels in this line via western blot as we were at the lower limit of sensitivity for our available dysferlin antibodies (data not shown), and so we did not pursue this line in further studies.

### Dysferlin 40aKO mice do not display a dysferlinopathy phenotype

**Fig. 1:**
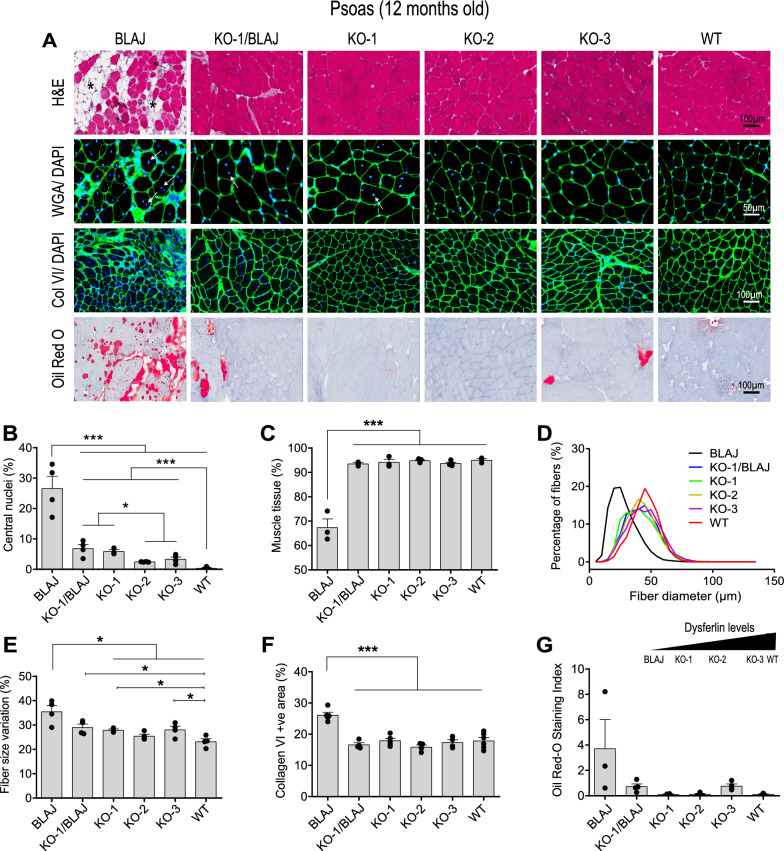
Knock out of dysferlin exon 40a does not result in a dysferlinopathy phenotype. **a** Representative images of skeletal muscle sections from the psoas of 12-month-old dysferlin exon 40aKO mouse lines, BLAJ and WT mice stained with haematoxylin and eosin (to display overall morphology), wheat-germ agglutinin (WGA)/DAPI (to highlight central nuclei, indicated by white and dark arrow heads), collagen VI/DAPI (to evaluate fibrosis) and oil-red O (to evaluate lipid content). Skeletal muscle from all 40aKO mice appear relatively normal compared to BLAJ muscle, without the overt inflammation, variable fiber size, fatty replacement (asterisk) and excess fibrosis seen in BLAJ mice. **b** Analysis of centralised nuclei in WGA/DAPI stained sections demonstrates a gradient of centralised nuclei inversely related to dysferlin protein expression. **c** Quantitative analysis of the percentage of muscle tissue across a whole muscle tissue section shows a reduction in muscle tissue in BLAJs only. **d** Frequency distribution of muscle fiber diameter size across the six different lines, showing predominance of smaller sized fibers in BLAJ mice and similar sized fibers between WT and 40AKO mice. **e** Coefficient of variation in fiber diameter in each of the six different lines expressed as a percentage. BLAJ show the greatest fiber size variation compared to WT and 40aKO mice **f** Quantification of collagen VI-stained area per field of view (700 µm x 500 µm). There was no significant difference between WT and 40aKO mice. Collagen VI is only elevated in BLAJ mice **g** Oil-red O-stained areas were quantified across the entire muscle section using the Scanscope digital slide analysis software. Three readings were recorded and averaged per animal. No significant difference in lipid content was detected between WT and 40aKO mice, whereas BLAJs showed a trend for increased lipid content. In all graphs, each dot represents an individual animal, n = 3–4 in **b-e** and G, n = 4–6 in **f**. Data are expressed as mean ± SEM. Differences were tested by One-way ANOVA with Tukey’s multiple-comparisons test. *p < 0.05, **p < 0.01, ***p < 0.001

To characterise the histopathology of 40aKO mice, serial cryosections were sampled from the gluteus, psoas, quadriceps and spinalis muscles at 12 months of age and compared to age-matched C57BL/6 wildtype (WT) and dysferlin-null BLAJ mice (the disease control line). Consistent with previous studies [16, 17], BLAJ mice displayed a dystrophic histopathology accompanied by inflammation, abundant central nuclei, fatty replacement of muscle tissue, interstitial collagen deposition, and variation in fiber size (Fig. 1, Additional files 3, 4, 5: Fig. S3A-5A). The extent of fat and collagen deposition was more pronounced in the psoas and gluteus muscles compared to the quadriceps and spinalis. Hematoxylin and eosin (H&E) stain of 40aKO muscle from all three lines revealed a relatively normal overall architecture with minimal overt dystrophic histopathology, in contrast to BLAJ mice (Fig. 1a). Notably, we also observed minimal dystrophic histopathology in muscles from 40aKO-1_low_/BLAJ crossed mice (40aKO-1/BLAJ) (Fig. 1a). In all four muscles examined, there was an inverse correlation between central nucleated fibers (CNF) and levels of dysferlin protein (Fig. 1b, Additional file 3, 4, 5: Fig. S3B-5B): dysferlin null BLAJ with ~ 27%—48% CNF fibers; 40aKO-1_low_ and 40aKO-1/BLAJ with ~ 5—12% CNF; 40aKO-2_mid_ and 40aKO-3_high_ with ~ 2—5% CNF; WT with ~ 0–4% CNF (Fig. 1b).

In all muscles except quadriceps, dysferlin-null BLAJ mice had significantly reduced mean fiber diameter (Fig. 1d, Additional file 3, 4, 5: Fig. S4D-5D) and subtle increase in fiber size variation (Fig. 1e, Additional file 3, 4, 5: Fig. S3–S5) compared to WT. In contrast, all 40aKO muscles showed broadly similar mean fiber diameter and fiber size variation as WT muscles, with only a subtle decrease in mean fiber diameter for 40aKO-1_low_ detected in psoas, the most severely affected muscle in BLAJ animals (Fig. 1d). This correlated with the significant reduction in muscle tissue cross sectional area in the psoas and gluteus of BLAJ mice, quantified from H&E-stained images (Fig. 1c, Additional file 4: Fig. S4C).

Oil-red O (ORO) staining showed a marked increase in fat deposition in psoas and gluteus muscles of aged dysferlin-null BLAJ mice, reproducibly attenuated in muscles from all age-matched 40aKO lines (Fig. 1a, g, Additional file 4: Fig. S4A and G). ORO staining of psoas muscle tissue from young mice at 18wks of age showed no evidence of excess lipid deposits in any of the samples examined including the disease control dysferlin-null BLAJ mice (Additional file 17: Fig. S8), suggesting fatty tissue replacement of myofibers occurs later in the dystrophic process in dysferlin-null BLAJ mice.

To further investigate the cellular location of ORO-stained fat deposits in aged (12 month) muscle tissue, we conducted Transmission electron microscopy (TEM) imaging on psoas cryosections from aged dysferlin-null BLAJ mouse, 40aKO-1_low_ mouse (the line with the least amount of dysferlin amongst our knockout lines) and control WT mouse. TEM analysis of the psoas muscle showed that most of the lipid in aged BLAJ muscle was predominantly adipocytes located between myofibers (Additional file 18: Fig S9). However, there was also evidence of intramyocellular lipid accumulation seen in a small proportion (~ 5%) of BLAJ myofibers (Additional file 18: Fig. S9). In contrast, the psoas muscle of WT and 40aKO-1_low_ line, similar to the ORO staining pattern, did not show conspicuous intermyofibrillar lipid deposits. Intracellular lipids were rarely sighted in WT and 40aKO-1_low_ myofibers and when present tended to be smaller and fewer in number compared to that of BLAJ myofibers (Additional file 18: Fig. S9). Furthermore, TEM analysis revealed that 40aKO-1_low_ myofibers appeared normal with well-aligned sarcomeres much like WT myofibers, whereas BLAJ myofibers exhibited several ultrastructural abnormalities previously reported by Grounds et al. [17], including misaligned sarcomeres (Additional file 18: Fig. S9).

Collagen VI staining showed a significant increase in collagen deposition in BLAJ mice (~ 30% increase relative to WT, *p* < 0.001, Fig. 1f, Additional file 4: Fig. S4F), also measurably attenuated in 40aKO mice which had similar levels to WT (Fig. 1f).

### Minimal amount of dysferlin is required to prevent disturbance of the lipidome

**Fig. 2:**
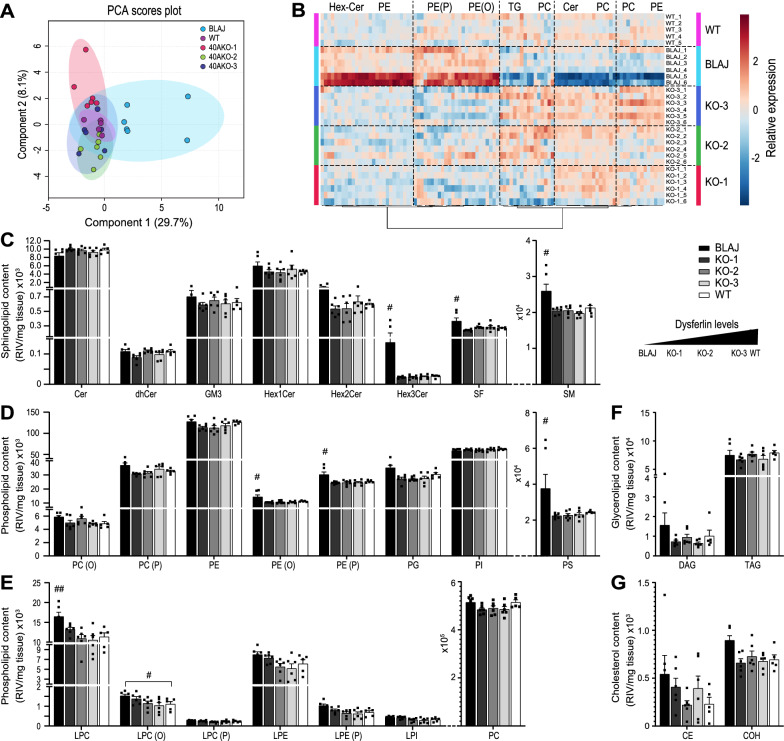
Dysferlin hypomorph lines lacking exon 40a maintain a normal skeletal muscle lipidome. **a** Lipidomics analysis was conducted on the quadriceps muscles of WT, BLAJ and the three 40aKO lines at 18wk of age. Principal component analysis (PCA) shows clustering of WT and 40aKO lines, and segregation of BLAJ samples. **b** Heatmap of the top 100 altered lipid species. Each row represents an individual animal. The predominant lipid subclass across multiple columns is shown on top of the graph. WT and 40aKO lines display a similar heatmap signature, distinct from BLAJ mice (**c**–**g**). Quantification of lipid species, expressed as raw intensity value (RIV)/mg tissue, showed normal sphingolipid (**c**), phospholipid (**d, e**), glycerolipid (**f**) and cholesterol **g** content in all three 40aKO lines, unlike BLAJ mice. Lipid species belonging to the same major lipid class (e.g. sphingolipids in **c**) have been graphed on the same axis. In all graphs, each dot represents an individual animal, n = 6/group. Data is represented as mean ± SEM. Differences were tested by One-way ANOVA with Tukey’s multiple-comparisons test. ^#^*p* < 0.05, ^##^*p* < 0.01. *Cer* ceramide; *dhCer* dihydroceramide; *G*_*M*_*3* G_M_3 ganglioside; *Hex1Cer* monohexosylceramide; *Hex2Cer* dihexosylceramide; *Hex3Cer* trihexosylceramide; *SF* sulphatide; *SM* sphingomyelin; *PC(O)* alkylphosphatidylcholine; *PC(P)* alkenylphosphatidylcholine; *PE* phosphatidylethanolamine; *PE(O)* alkylphosphatidylethanolamine; *PE(P)* alkenylphosphatidylethanolamine; *PG* phosphatidylglycerol; *PI* phosphatidylinositol; PS = phosphatidylserine; *LPC* lysophosphatidylcholine; *LPC(O)* lysoalkylphosphatidylcholine; *LPC(P)* lysoalkenylphosphatidylcholine; *LPE* lysophosphatidylethanolamine; *LPE(P)* lysoalkenylphosphatidylethanolamine; *LPI* lysophosphatidylinositol; *PC* phosphatidylcholine; *DAG* diacylglycerol; *TAG* triacylglycerol; *CE* cholesteryl ester; *COH* cholesterol

Lipidomic analyses of quadricep muscles of 40aKO lines, WT and BLAJ mice was performed at 18-wks of age, prior to conspicuous lipid deposits in disease control BLAJ mice (Additional file 3: Fig. S3A, upper panel, Additional file 17: Fig. S8) [15, 16] and enabling comparison with previously published lipidomics data in BLAJ mice [15]. Figure 2a principal component analysis (PCA) shows segregation of BLAJ specimens from WT and 40aKO samples, with two of the six BLAJ samples outliers (Fig. 2a). These samples were from the same litter as the other four animals and showed an insignificant trend for increased lipid content relative to the other four BLAJ samples (Fig. 2b–g). All 40aKO specimens clustered with WT samples, indicating a similar lipidome to the WT samples (Fig. 2a). Figure 2b represents a heatmap of the top 100 significantly altered lipid molecular species. Consistent with the PCA plot and in line with results from previous studies [15], BLAJ muscle shows distinctive disturbances in lipidomic profile. The lipidomic mass spectrometry in this study assessed 372 lipid species categorised into four main lipid classes present in skeletal muscle; sphingolipids, phospholipids, glycerolipids and cholesterol (Fig. 2c–g) [15]. In all lipid classes and subclasses assessed, the three 40aKO lines were similar to each other and not significantly different to WT samples. In contrast, BLAJ muscles relative to WT and 40aKO samples, showed significant elevation in several lipid species notably trihexosylceramides (Hex3Cer, ~ sixfold, *p* < 0.05), phosphatidylserines (PS, ~ twofold, *p* < 0.05), sulfatides (SF, ~ 1.3-fold, *p* < 0.05) and sphingomyelin (SM, ~ 1.3-fold, *p* < 0.05) (Fig. 2c).

### Expression levels of dysferlin-associated proteins, contractile, structural and lipid metabolism proteins correlate with levels of dysferlin in 40aKO mice

**Fig. 3:**
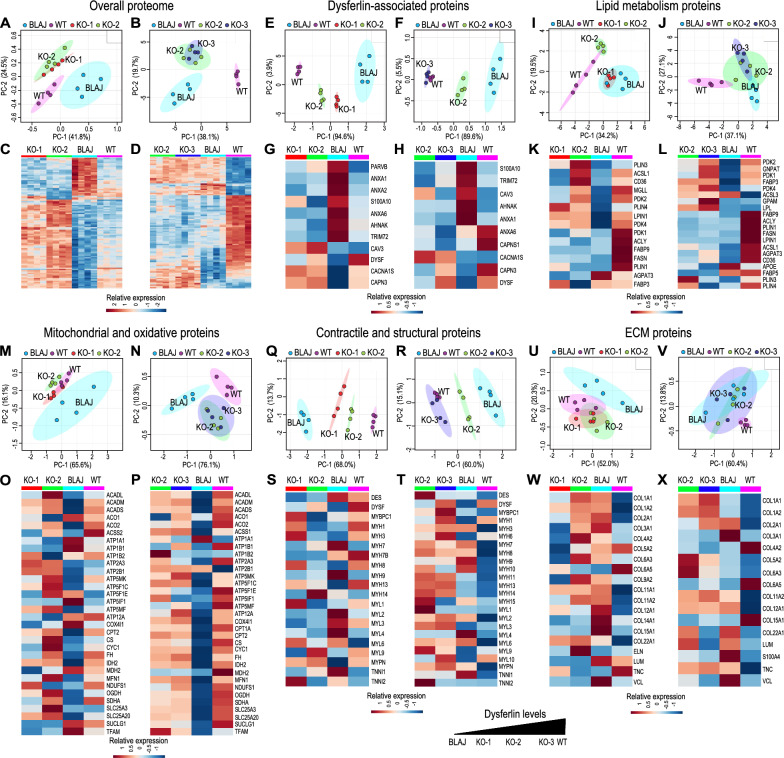
Proteomics analysis of Dysferlin Exon 40a knockout mice. Quadriceps muscle sections from three *dysf40a* KO lines expressing variable amounts of dysferlin protein (KO-1_low_, KO-2_mid_ and KO-3_high_, all aged 18 weeks) were subjected to LC–MS/MS for proteomics analysis alongside age-matched control WT and dysferlin-null BLAJ mice. Samples were probed across two different experiments, with the first set comprising KO-1, KO-2, WT and BLAJ (left side panels) and the second set comprising KO-3, KO-2, WT and BLAJ (right side panels), n = 4 samples/group. For each of the six categories of proteins listed in the figure, a principal component analysis (PCA) graph has been plotted with the corresponding heatmap expression panel displayed directly below it, to highlight differences and similarities between groups. **a**, **b** PCA plot of all proteins analysed shows that KO lines have a similar overall proteomic profile that is distinct from that of WT and BLAJ, which constitute the greatest phenotypic differences. **(C, D)** Heatmap of the top 100 altered proteins showed similar trends to the PCA plots in (**a**) and (**b**). **e**–**h** PCA and heatmap analysis of common dysferlin-associated proteins shows vast differences between WT and BLAJ and a dysferlin-dose dependent effect on expression of these proteins in KO lines. **i**–**l** Analysis of lipid metabolism proteins also showed a dysferlin-dose dependent effect, KO1_low_ clustered with BLAJ, KO2_mid_ showed partial segregation from BLAJ while KO-3_high_ was well segregated from BLAJ. **m**–**p** Expression patterns of mitochondrial and oxidative proteins were similar to lipid metabolism proteins except, KO lines were more similar to WT than BLAJ. **q**–**t** Expression of contractile and structural proteins mirrored the expression pattern of dysferlin-associated proteins (**e**–**h**). **u–x** Analysis of ECM proteins showed minimal segregation (differences) between WT, BLAJ and KO lines. ANOVA post-hoc statistical analysis for each of the heatmap expression panels can be found in Supplementary Tables 3–7. For all heatmap data displayed, conventional protein/gene abbreviations have been used which can be searched in online protein databases or in Supplementary Table 2 for reference

Proteomics studies were performed on the contralateral quadriceps of a subset of the same 18wk old 40aKO, WT and BLAJ mice used for the lipidomics analysis (n = 4/group) to predate significant lipid and fibrotic alterations in muscle and to allow direct correlation of proteomics and lipidomics findings. Samples were run in two separate experiments because the LC–MS/MS method used could only accommodate 16 samples per run. Experiment #1 consisted of WT, 40aKO-1_low_, 40aKO-2_mid_ and BLAJ samples. Experiment #2 consisted of WT, 40aKO-2_mid_, 40aKO-3_high_ and BLAJ samples. Lack of dysferlin in BLAJ mice resulted in a distinct proteomic profile from WT mice (Fig. 3a–d), with disturbances noticed in proteins associated with various cellular locations and functions (Additional file 6: Fig. S6, Additional file 9: Table 2). The overall proteomic profile of 40aKO mice was intermediate between WT and BLAJ with no clear correlation between the amount of dysferlin present and the level of proteomic disturbance (Fig. 3a–d). However, when we looked more closely, we noticed that expression of dysferlin-associated proteins (Fig. 3e–h, Additional file 10: Table 3) as well as contractile and structural proteins (Fig. 3q–t, Additional file 12: Table 5) directly correlated with the amount of dysferlin expressed in 40aKO mice. The 40aKO-3_high_ line (~ 90% dysferlin) closely clustered with WT samples (Fig. 3f, r), 40aKO-2_mid_ (~ 50% dysferlin) displayed an intermediate phenotype between WT and BLAJ (Fig. 3e,f,q,r), and the phenotype of 40aKO-1_low_ (~ 10–20% dysferlin) samples was intermediate between 40aKO-2_mid_ and BLAJ (Fig. 3e, q). In contrast, the overall ECM phenotype of BLAJ mice was not substantially different from that of WT and 40aKO lines (Fig. 3u–x, Additional file 13: Table 6). Expression of lipid metabolism proteins (Fig. 3i–l, Additional file 11: Table 4) including mitochondrial and oxidative proteins involved in fatty acid β-oxidation (Fig. 3m–p, Additional file 14: Table 7), was somewhat dependent on the dose of dysferlin but not the same extent as dysferlin-associated proteins. GO and KEGG pathway analyses further confirmed disturbance of lipid metabolism in BLAJ mice but not 40aKO mice, which were more like WT (Additional file 6: Fig. S6).

### Lack of Dysferlin exon 40a does not impair membrane repair

**Fig. 4:**
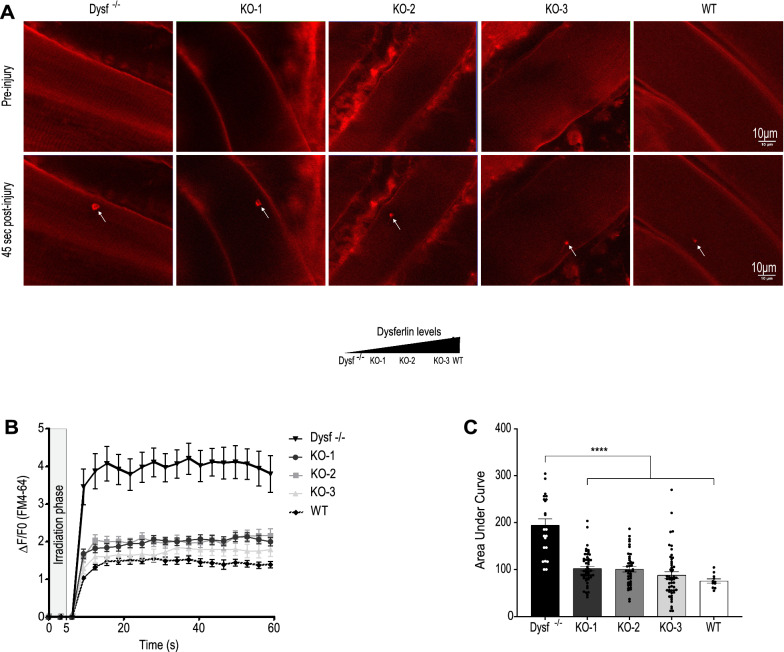
Expression of ~ 10–20% of WT dysferlin protein levels is sufficient for normal membrane repair. **a** Representative images showing time-dependent accumulation of FM-64 dye following laser-induced membrane damage of flexor digitorum muscle fibers (FDBs) in whole FDB muscle harvested from 40aKO mice (KO-1-to-KO-3), WT and dysferlin-null mice aged 4–6 months. White arrows point to the site of irradiation at 45 s post injury. **b** Comparison of FM-64 dye curves of each line, gathered from images captured at 3 s intervals, including the duration of irradiation (gray area), until 60 s. FM-64 dye begins to rise as soon as irradiation is complete at 5 s. **c** Quantification of the area under the curve (AUC) of each line in (**a**). There is no significant difference in the average AUC of 40aKO mice and WT, indicating normal membrane repair capacity in all 40aKO lines. In contrast, the average AUC of dysferlin null fibers is at least twice that of WT and 40aKO, indicative of defective membrane repair. Dots in graph (**b**) represent numbers of FDBs assessed per genotype, Dysf.^−/−^ = 22, KO-1 = 50, KO-2 = 42, KO-3 = 51, WT = 11. Data is represented as mean ± SEM. Differences were tested by One-way ANOVA with Tukey’s multiple-comparisons test. ***p < 0.001

Flexor digitorum brevis (FDB) fibers from young adult 40aKO, WT and BLAJ mice (aged 6 months) were subjected to UV laser-induced damage of the sarcolemma in the presence of extracellular calcium and FM4-64 dye. As observed in previous studies [6], following UV-laser damage, muscle fibers from wild type mice promptly repaired UV-laser induced membrane breaches resulting in low FM4-64 dye entry. In contrast, dysferlin null mice showed increased intracellular accumulation of FM4-64 dye due to defective membrane repair (Fig. 4a–c). Surprisingly, there was no significant difference in membrane repair compared to WT in all three 40aKO lines (Fig. 4a–c). There was a slight trend for increased dye accumulation with reduced amount of dysferlin protein among the 40aKO mice (Fig. 4b). However, quantification of the area under the time course curves did not determine this to be statistically significant. These results provide empirical evidence that calpain-cleavage within exon 40a is not essential for muscle membrane repair and 10–20% of dysferlin protein as present in the 40aKO-1 line is sufficient to maintain normal membrane repair (Fig. 4c).

## Discussion

In this study, we employed CRISPR/Cas-9 gene editing in mice to knock-out dysferlin exon 40a, to preclude expression of the ‘calpain cleavable’ forms of dysferlin. Alterations in *Dysf* pre-mRNA splicing incidentally created three 40aKO lines expressing varying levels of dysferlin (without residues encoded by exon 40a), allowing us to simultaneously evaluate the role of exon 40a and hypomorphic levels of the canonical dysferlin isoform (NM_001077694.2) on the muscle lipidome, proteome, membrane repair and development of a muscular dystrophy. Our collective results show that loss of expression of dysferlin isoforms encoding exon 40a does not measurably impact membrane repair capacity of isolated muscle fibers and does not result in detectable lipid or protein imbalance nor result in a dystrophic histopathology. Critically, our collective results indicate that ~ 10–20% of normal dysferlin expression is sufficient for normal membrane repair capacity, prevents the lipidome/proteome imbalance and the muscular dystrophic phenotype of dysferlin-null BLAJ mice.

Detailed histopathological analyses demonstrated an inverse correlation between CNF counts and levels of dysferlin protein expression, indicating that the more dysferlin present, the less susceptible myofibers are to degeneration and regeneration. Our mouse models support previous findings that low level expression of dysferlin can ameliorate dystrophic pathology. Sinnreich et al. [35] reported an individual affected with dysferlinopathy with compound heterozygous *DYSF* variants (NM_001130987.2(DYSF):c.4873del, c.4876G > C and c.3497-33A > G). C.3497-33A > G disrupts an intron 31 branchsite, resulting in in-frame skipping of exon 32 and expression of ~ 10% of normal dysferlin levels. The proband shows elevated creatine kinase (25 times normal) and mild weakness. In addition, Llanga et al. [36] and Lostal et al. [37] have previously demonstrated that adeno-associated viral vector (AAV) administration of dysferlin in pre-dystrophic BLAJ mice, resulted in expression of ~ 10% of WT dysferlin levels in skeletal muscle tissue and improved muscle integrity, with similar histopathological improvements reported in our study. However, our mouse models are most similar to the clinical study by Sinnreich et al. [35], with endogenous dysferlin expression at low levels maintaining muscle health and function rather than exogenous dysferlin expression reversing pathology.

Disrupted membrane repair is one of the pathological hallmarks of the murine model of dysferlinopathy [11]. We showed that all three 40aKO lines did not demonstrate defective membrane resealing despite lacking exon 40a. Approximately 10–20% of total dysferlin expressed in the 40aKO-1 line was sufficient to produce normal membrane resealing to restore barrier function and stop dye influx into muscle fibers. This finding indicates that *Dysf* exon 40a and minidysferlin_C72_ are dispensible for acute membrane resealing following laser-induced membrane disruption. However, *Dysf* exon 40a transcripts and minidysferlin_C72_ could function in other cell functions such as vesicle trafficking, and confer protection in other forms of mechanical injury such as osmotic shock as previously reported in human myotubes by Ballouhey et al. [34].

It is well established that absence or significantly reduced levels of dysferlin contributes to defective membrane repair [11] and lipid accumulation in dysferlinopathic tissues [15, 17]. In the current study we show for the first time that only a small amount of dysferlin (~ 10–20%) was sufficient to maintain a WT phenotype and prevent early dysregulation of the lipidome seen in BLAJ mice (Fig. 2). Haynes et al. [15] previously report transcriptional changes that contribute to alterations in the lipid profile of similarly aged BLAJ mice. Herein, we corroborate this data at a protein level with multiple lines of evidence that shows both lipid composition and proteins involved in lipid biosynthetic pathways and metabolism are influenced by the dose of dysferlin (Fig. 3).

The precise mechanism through which dysferlin influences the muscle lipidome remains uncertain. Our collective studies of truncated dysferlin isoforms [33] and of different ferlin isoforms [8] are most consistent with the notion that vesicles decorated by dysferlin and myoferlin may integrate into the target membrane, whereas vesicles decorated by otoferlin and Fer1L6, or with C-terminal fragments of dysferlin containing one or two C2 domains and the transmembrane domain, are quickly endocytosed, suggesting they do not integrate. We speculate the Fer/DYSF region of dysferlin and myoferlin, absent in otoferlin and Fer1L6 (and removed in C-terminal dysferlin fragments), plays a key role in the integration of dysferlin vesicles into the target membrane to enable lipid and protein components to be incorporated into this target membrane—with dysferlin’s numerous C2 domains implying complex regulation of target membrane recognition, binding and fusion. We further postulate that dysferlin regulates a non-redundant vesicular trafficking pathway that delivers specific lipids and proteins to the sarcolemma and luminal contents to the extracellular matrix. We suspect that disturbance of routine replacement of specific sarcolemmal lipids may conceivably be the upstream lynchpin driving pathology in dysferlinopathy. When dysferlin is absent, these specific vesicles can’t be delivered to the place they need to be by anything else. Maintenance of the muscle membrane lipidome goes awry, signalling pathways that depend on specific lipid microdomains go awry and signals are elicited to upregulate lipid delivery—with defective membrane repair a downstream biomarker of collective lipid and protein abnormalities.

The clear difference in the proteomic profile between WT and BLAJ muscle at 18 weeks of age show that proteomic disturbance precedes development of a dystrophy. In aged dysferlin null mice and individuals with dysferlinopathy, lack of dysferlin alters expression of many dysferlin-associated proteins including AHNAK, Annexin A2, Calpain-3, Caveolin-3 and MG53 [9, 38, 39]. Data from the current study indicates that perturbation of dysferlin-associated proteins occurs early and correlates broadly with hyopmorphic dysferlin levels. Interestingly, expression of contractile and structural proteins seems more influenced by the amount of dysferlin expressed, compared to ECM proteins.

In summary, collective findings from this study show that *Dysf* exon 40a is dispensible and expression of ~ 10–20% of dysferlin protein is sufficient to prevent early dysregulation of the muscle lipidome and proteome, maintain membrane repair capacity and prevent the overt dystrophy seen in aged dysferlin null BLAJ mice. Though one cannot directly infer results from a mouse model to a human, our findings provide great optimism for future gene therapy trials aimed at restoring levels of the canonical dysferlin isoform in the skeletal muscles of affected individuals, indicating only low levels of dysferlin may result in a clinically meaningful improvement in symptomology. There is now great need to gain deeper insight into the earliest disruptions to the sarcolemmal lipidome and determine whether this precedes, or occurs concurrently with, proteomic disturbances.

## Materials and Methods

### CRISPR-Cas-9 gene editing, PCR and genotyping

Using the CRISPR design tool at http://crispr.mit.edu we designed CRISPR RNA guides targeting the exon 40a locus of murine dysferlin. PAM sequence is shown in Additional file 1: Fig. S1A. To avoid issues with off-target effects, we chose to use the paired guide/Cas-9 nickase strategy when creating our mouse model. Guide RNA and Cas-9n mRNA were co-injected into pronuclear C57BL/6 embryos and the resultant pups were screened by PCR across the exon 40a locus. PCR primers (Sigma) used for genotyping are shown in Additional file 1: Fig. S1A and Additional file 8: Table 1. The 1 × PCR mix constituted 15µ L of 2 × Buffer D (Astral Scientific), 12 µL milli-Q water, 1 µL forward primer, 1 µL reverse primer, 0.2 µL Taq DNA polymerase (Invitrogen) and 1 µL of template gDNA (~ 500 ng). PCR cycling conditions were as follows: (i) 95 °C–3 min, (ii) 30x (95 °C –30 s, 58 °C –30 s, 72 °C -1 min), (iii) 72 °C -5 min. PCR products were mixed with 5 × DNA loading buffer (Bioline Meridian Bioscience) and run alongside a 100 bp ladder (Bioline Meridian Bioscience) on 1.8–3% TAE gel at 100 V for ~ 1 h. Founder mice carrying identifiable gene-editing events in and around exon 40a were consequently bred to homozygosity so RNA and protein could be studied without the confounding effect of the normal/WT suite of dysferlin transcripts and isoforms. We generated three founder mice that each carried a different deletion in and around exon 40a (Additional file 1: Fig. S1) and derived a distinct mouse line from each founder. For further details on generation and characterisation of exon 40a knockout mice, refer to Additional file 7.

### SDS PAGE and Western blotting

Western blotting was done as previously described [26, 39]. Briefly, muscle sections (10 mm^2^, 8 µm thick) were homogenised on ice in SDS lysis buffer (2% SDS, 125 mM Tris pH 7.5) supplemented with protease inhibitors (Sigma Aldrich). Twenty micrograms of protein was mixed (1:1) with 5X SDS loading buffer, heated at 95 °C for five minutes and then resolved on a 3–8% Tris–acetate polyacrylamide gel (Thermo Fisher Scientific Melbourne, VIC, Australia) alongside the Page Ruler™ Plus Protein Standard (Thermo Fisher Scientific). Samples were then transferred onto a polyvinylidene fluoride (PVDF) membrane (Merck Millipore, Bayswater, WA, Australia). After blocking the membrane in 5% skim milk/PBS/0.1% Tween-20 for 1 h, anti-dysferlin primary antibody (1 in 500, NCL Hamlet-CE, Leica Biosystems,Buffalo Grove, IL) or anti-GAPDH loading control (1 in 2000, Millipore Bioscience Research Reagents) was incubated overnight at 4 °C with shaking. The membrane was washed in PBS/0.1% Tween-20 prior to incubation in a species-specific horseradish peroxidase conjugated secondary antibody for 2 h at room temperature (R.T). The membrane was washed again, and the signal developed on an X-ray film following addition of a chemiluminescence substrate (GE Healthcare, Parramatta, Australia).

### Immunoprecipitation of dysferlin and calpain cleavage

Immunoprecipitation was conducted as previously described by Redpath et al. [22] with minor modifications. Briefly, quadriceps muscle sections from WT, 40aKO lines 1-to-3 and BLAJ mice were lysed in RIPA buffer without calcium and supplemented with protease inhibitors. Endogenous dysferlin was immunoprecipitated with N-terminal rabbit polyclonal antibody Romeo (Epitomics Inc.) and protein G–sepharose beads (GE Healthcare) overnight at 4 °C. Dysferlin-bound sepharose beads were then either incubated with 20 active unit (A.U.) of purified recombinant calpain-2 at 30 °C for 3 min or left undigested, in the presence of 2 mM CaCl_2_. Digestion was quenched by reconstitution into SDS lysis buffer (2% SDS, 10% glycerol, 50 mM Tris, pH 7.4, and 10 mM dithiothreitol (Sigma-Aldrich). Samples heated to 94 °C for 3 min and dysferlin was detected by Western blot analysis with the C-terminal antibody Hamlet-1.

### Histopathology

#### Cryosectioning

For histopathology analysis, gluteus, psoas, quadriceps and spinalis muscles were dissected from ~ 12 months old BLAJ, Dysferlin exon 40aKO and C57BL-6 WT mice. Muscles were frozen for 30 s in 2-methylbutane (Sigma Aldrich, Australia) cooled in liquid nitrogen. Samples were then stored at − 80 °C for future use. Transverse muscle sections (8 µm) were collected onto Superfrost Plus glass slides (Menzel-Glaser, Braunschweig, Germany) using the cryostat CM1950 (Leica Biosystems).

#### Haematoxylin and Eosin (H&E) and Oil Red O Staining

H&E and Oil-Red O histochemical staining on frozen muscle sections was performed as previously described by Sullivan et al. [40]. Images were acquired using the Aperio ScanScope CS (Leica Biosystems) at × 20 magnification and visualised using the Aperio Image Scope companion software (version 11.2.0.780, Lecia Biosystems). The Aperio Image Scope ‘Colour Deconvolution v9’ algorithm was used to analyse the percentage of red stained (Oil-Red O positive) areas in each muscle section. However, it must be noted that Oil-Red O staining on cryosections is confounded by smearing of lipids across the section, which makes it difficult to quantify and to determine whether lipid droplets were localised in myofibers or adipocytes [17]. To help overcome this caveat, the staining intensity and area of Oil-Red O-stained areas was accounted for to calculate the staining index.

#### Wheat-Germ Agglutinin (WGA)/DAPI Staining

To assess the presence of centralised nuclei in muscle sections, the sarcolemma was stained with WGA Alexa Fluor™ (AF)-488 conjugate (Thermo Fisher Scientific, Australia) and DAPI (1 µg/ml PBS, Thermo Fisher Scientific, Australia) was used to stain nuclei. Tissue sections were fixed in ice-cold 4% paraformaldehyde (PFA) for 10 min, washed twice for three minutes in PBS and then blocked in 2% BSA/PBS for 30 min prior to incubation in WGA-AF488 (diluted 1 in 200 Hank’s Buffered Saline Solution) for 10 min at R.T. Slides were washed three times for five minutes in PBS, counterstained with DAPI for five minutes then washed again in PBS before mounting and coverslipping in FluorSave™ Reagent. Non-overlapping images covering the entire muscle section were captured at × 10 magnification using the Leica DMi8 microscope (Leica Microsystems). A custom written Image J macro (Additional file 7) was used to enumerate centralised nuclei. Briefly, for every fiber, the macro detected its outline, measured the size of the fiber (area and minimum Feret diameter), then shrunk it slightly to exclude peripheral nuclei before counting the number of central nuclei.

#### Collagen VI and Dysferlin immunofluorescent staining

Following fixation in ice-cold 4% PFA for 10 min, slides were washed in PBS, fixed in 1:1 methanol/acetone for 10 min then blocked in 2% BSA/PBS for 30 min. For dysferlin staining, prior to fixation, muscle sections were subjected to heat mediated antigen retrieval as previously described by Roche et al. [41]. In addition, slides were blocked with goat-anti-mouse Fab fragments (1 in 20, Jackson ImmunoResearch Laboratories) for 2 h at R.T or overnight at 4 °C, prior to the addition of primary antibody. Slides were incubated in primary antibody for 2 h at R.T or overnight at 4 °C. Collagen VI was detected using 1 in 2000 affinity purified rabbit polyclonal collagen type VI antibody (Fitzgerald Industries International), dysferlin was detected using NCL-Hamlet-1 (1 in 50). Following primary antibody incubation, slides were washed three times for five minutes in PBS, blocked in 2% BSA/PBS for 10 min then incubated in secondary antibodies goat anti-rabbit AF-488 or donkey anti-mouse AF-488 (1 in 200, Thermo Fisher Scientific) for 2 h at R.T. Slides were washed in PBS, counterstained with DAPI (1 µg/ml PBS) for five minutes (and WGA-AF555 when staining for dysferlin), washed again in PBS and finally mounted and coverslipped in FluorSave™ Reagent. Images were acquired on the DMi8 microscope under × 20 and × 40 objectives. The percentage of collagen VI positive stained areas was estimated using a custom written macro in Image J (Additional file 7).

#### Transmission electron microscopy imaging

Muscles previously frozen in 2-methylbutane were removed from the -80 freezer, cut into ~ 2mm^2^ transverse cross sections at -20 °C and immersed in cold 2.5% glutaraldehyde in 0.1 M cacodylate buffer overnight. Samples were washed in 0.1 M cacodylate buffer and post fixed with 2% osmium tetroxide for 2 h, dehydrated in a series of ethanol and embedded in TAAB Low Viscosity Resin (TAAB Laboratories). Sections were cut at 90 nm using a UC7 ultramicrotome (Leica Microsystems), mounted on formvar coated nickel grids, and stained with 2% uranyl acetate in 50% ethanol (10 min) and Reynold’s lead citrate (4 min). Grids were examined with a Jeol-1400 transmission electron microscope (JEOL Ltd., Tokyo, Japan) and micrographs recorded using a Jeol sCMOS Flash camera.

### Lipidomics

Lipidomics analysis was conducted on the quadriceps muscle frozen on dry ice after harvest from 18wk old 40aKO lines, WT and BLAJ mice. Approximately 20 mg of muscle tissue from the mid-belly of the quadriceps was sampled from each mouse and shipped on dry ice to Metabolomics Australia at The University of Melbourne. To prepare samples for lipid extraction and LC–MS/MS analysis, 20 mg of muscle tissue was placed in a cryomill tube and 600 µL of chilled methanol:chloroform (9:1) was added, including internal standards. Samples were homogenised using the cryomill set at 6800 rpm for 3 × 30 s cycles. Homogenates were transferred into new 2 mL Eppendorf tubes and 680 μL of 100% chloroform was added and thermomixed at 1000 rpm/20 °C for 15 min. Samples were centrifuged at 15,000 rpm at 0 °C for 10 min and then transferred to fresh 2 mL Eppendorf tubes before being placed in a speedvac to allow supernatants to evaporate to complete dryness. Finally samples were reconstituted in 100 µL of water-saturated butanol:methanol (9:1). Lipid analysis was done using the LCMS_C18_MRM (positive mode) method on the Agilent QQQ 6490 mass spectrometer. Agilent Mass Hunter QQQ Quant was used to quantify individual lipid species. Statistical data analysis and data normalisation was further carried out in MetaboAnalyst 5.0 (https://www.metaboanalyst.ca/docs/Tutorials.xhtml). Data was normalised by median log transformation with mean centering and no filter applied prior to generating PCA curves and heatmaps from the.cvs file of raw intensity values.

### Proteomics

Proteomic analysis was conducted on the contralateral quadriceps muscles of 18wk old 40aKO lines, WT and BLAJ mice used for lipidomics analysis. Muscles were frozen in 2-methylbutane after harvest. To prepare samples for proteomic analysis, 10 muscle sections (10mm^2^, 8 µm thick) per animal were homogenised on ice in 200 µL of 2%SDS/50 mM TEAB lysis buffer (pH 7.5) with protease inhibitors. After adding lysis buffer, samples were vortexed and then sonicated three times, cooling the samples on ice after each round of sonication. Samples were then frozen on dry ice and sent to the Proteomics facility at The Children’s Medical Research Institute (CMRI). LC–MS/MS analysis of muscle tissue lysates was performed on Dionex UltiMate 3000 RSLC nano system and Q Exactive Plus hybrid quadrupole-orbitrap mass spectrometer (Thermo Fisher Scientific) as described in supplementary methods. The resulting proteomics raw data was subjected to the Remove Unwanted Variation (RUVIII) method [42] from the RUV R package [43] to minimise batch-batch experimental variation, as further explained in Supplementary material. The processed raw LC–MS/MS data was then further analysed using MetaboAnalyst 5.0 (https://www.metaboanalyst.ca/docs/Tutorials.xhtml). Data was normalised by mean centering with no filter applied before generating PCA curves and heatmaps from the.cvs file of log transformed values.

### Membrane repair assay

A cohort of 40aKO mice were shipped to the Ohio State University in the United States, for membrane repair assays (M20_1074). FDB muscles were surgically isolated from 40aKO mice, including age (4–6 months old) and sex-matched dysferlin null and WT control mice, according to protocols previously published by Guschina et al. [6]. Briefly, intact FDB muscles were dissected at the tendons from animals and attached to 35 mm glass-bottom imaging dishes (MatTek) with liquid bandage (New-Skin). Muscles were surrounded with Tyrode’s solution containing 2.0 mM Ca2 + and 2.5 μM FM4-64 amphiphilic fluorescent styryl pyridinium dye (Life Technologies). An Olympus FV1000 multi-photon laser scanning confocal system was used to irradiate the cell membrane and induce a focal injury. A circular regional of interest on the cell membrane was selected for irradiation at 20–30% laser power for 5 s. Full field images were captured every 3 s, continuing for 60 s. Fluorescence intensity at the injury site in these images was determined using ImageJ software (NIH). Cell membrane repair kinetics were measured by establishing the change in fluorescence intensity (ΔF/F0). These values were calculated using the following equation for each image: (injury area fluorescence-background area fluorescence)/background fluorescence. To preclude potential experimental bias, all experiments were performed in a blinded fashion.

### Statistics

Statistical data analysis was performed using Graph-Pad Prism 9 software. Differences between genotypes was evaluated by One-Way ANOVA followed by Tukey’s multiple comparison test. Pairwise analysis was done using unpaired student t-test. P-values less than 0.05 were considered significant. Graphed data is represented as mean ± standard error of the mean (SEM).

## Supplementary Information

**Additional file 1. Fig. S1.** Genetic characterisation of dysferlin exon 40a knockout mice following CRISPR/Cas-9 genome editing. **(A) **Microdeletion events in and around dysferlin exon 40a in the three dysferlin exon 40a knockout mice generated by CRISPR/Cas-9 gene editing. 40aKO-1 carries a 24 bp deletion preceding the splice acceptor. 40aKO-2 carries a 65 bp deletion spanning intron 40 and exon 40a. 40aKO-3 carries an in-frame 12 bp deletion within exon 40a. Sanger sequencing primers flanking exon 40a (red text) were used to determine the precise genomic variations induced by CRISPR/Cas-9 activity. **(B) **PCR screening of genomic DNA from WT and dysferlin exon 40a knockout mice using primers that flank exon 40a (red text). PCR product sizes are indicated on the gel **(C)** PCR amplification of cDNA derived from the skeletal muscle and the lung of a WT and exon 40aKO-1 homozygous mouse. Using an upstream cryptic acceptor splice site (black arrowhead in **A**), ectopic intron 40 sequence and exon 40a is more frequently spliced into 40aKO-1 *Dysf* transcripts relative to exon 40a inclusion into *Dysf* transcripts of WT mice. 40aKO-1 transcripts utilising the upstream cryptic acceptor (black arrowhead in **A**) encode a premature termination codon (PTC) and should thus be subject to nonsense-mediated decay (NMD). **(D)** PCR amplification of cDNA derived from the skeletal muscle of three exon 40aKO-2 mice and one WT mouse. The upstream cryptic acceptor (black arrowhead in **A**) is also utilised in 40aKO-2 mice to splice in ectopic intron 40 sequence and exon 40a into *Dysf* transcripts more frequently than exon 40a is spliced into *Dysf* transcripts of WT mice. These transcripts (upper band in 40aKO-2) encode a PTC and should thus be subject to NMD. **(E) **Dysferlin exon 40aKO-3 mice exclusively express *Dysf* transcripts lacking exon 40a. These transcripts are in-frame and predicted to encode full length dysferlin. In skeletal muscle, there is a minor *Dysf* transcript expressed that lacks exon 41.

**Additional file 2. Fig. S2.** Localisation and quantification of dysferlin protein expression in dysferlin exon 40a knockout mice generated by CRISPR/Cas-9 genome editing. **(A) **Immunofluorescent staining for dysferlin using NCL Hamlet-1 on quadriceps muscle sections from 12-month-old WT, dysferlin 40aKO and BLAJ mice. Dysferlin is predominantly expressed at the sarcolemma but is reduced to varying degrees in the 40aKO lines compared to WT. Images representative of n = 3/group. **(B) **Western blot of protein extracted from quadriceps, psoas major, heart and spinalis muscles indicated that dysferlin protein levels in *Dysf* 40aKO lines (abbreviated KO-1-to-3) relative to WT are as follows: ~10-20% in KO-1, ~50% in KO-2 and ~90-100% in KO-3. (**C**) Proteomic analysis of dysferlin protein levels in quadriceps muscles of WT, BLAJ and exon 40aKO mice (n = 4/group), revealed similar trends to semiquantitative estimates by Western blot **(B)**, except for higher dysferlin estimates in BLAJ mice (~10-25% of WT), particularly in experiment 1. Graphed data is represented as mean ± SEM.

**Additional file 3. Fig. S3.** Histopathological analysis of quadriceps muscles of 40aKO mice. **(A)** Haematoxylin and eosin stain of young 18 wk old mice, including BLAJ mice, shows lack of overt dystrophic features (centralised nuclei, fibro-fatty deposits) in any of these mice. The H&E image of 18wk old KO-1/BLAJ has intentionally been omitted because we did not have samples from this line at this age. Compared to aged (~12 months) BLAJ mice, age-matched 40aKO mice do not display signs of overt dystrophy. **(B) **Quantification of central nucleated fibers (CNF) from WGA/DAPI stained images shows significantly reduced CNF counts in 40aKO mice compared to BLAJs. CNF counts of KO-2 and KO-3 are similar to that of WT. KO-1 and KO-1/BLAJ have slightly elevated CNF counts relative to WT. There is no significant difference between 40aKO lines, WT and BLAJ muscle tissue cross-sectional area **(C)**, fiber diameter size frequency distribution **(D)** and oil-red O staining index (lipid content, **F**, however, there is significant fiber size variation in BLAJ mice **(E)**. In all graphs, each dot represents an individual animal, n = 3-4/genotype, data is represented as mean ±SEM, differences between groups were assessed by One-way ANOVA followed by Tukey’s multiple comparison test.

**Additional file 4. Fig. S4.** Histopathological analysis of gluteus muscles from aged 40aKO mice. **(A) **Representative images of gluteus muscle sections from 12-month-old 40aKO mice, WT and BLAJ stained with H&E (morphology), WGA/DAPI (CNF counts), Col VI/DAPI (fibrosis) and oil-red O (fat deposition) evaluation. 40aKO mice are histologically similar to WT, lacking overt dystrophic features seen in BLAJ mice. **(B)** CNF count is inversely proportional to the amount of dysferlin expressed. KO-2 and KO-3 are not significantly different from WT. KO-1 and KO-1/BLAJ CNF counts are slightly but significantly elevated relative to KO-2 and KO-3. BLAJs have the highest CNF count, at least 5-fold more than KO-1. Quantitative analysis of muscle tissue cross-sectional area **(C)**, fiber diameter frequency distribution **(D)** and size variation **(E)**, collagen content **(F)** and fat deposition **(G)**, revealed no significant differences between 40aKO lines and WT, in contrast to BLAJs which showed significant alterations in all these parameters. In all graphs, each dot represents an individual animal, n = 3-4 in **B-E** and **G**, n = 4-5 in **F**. Data are expressed as mean ± SEM. Differences were tested by One-way ANOVA with Tukey’s multiple-comparisons test. *p<0.05, **p<0.01, ***p<0.001.

**Additional file 5**. **Fig. S5.** Histopathological analysis of spinalis muscles from aged 40aKO mice. **(A) **Muscle sections from spinalis of 12-month-old dysferlin 40aKO mice, WT and BLAJ mice were stained with H&E, WGA/DAPI and oil-red O to assess their morphology, presence of centrally nucleated fibers (CNF) and fatty deposition respectively. **(B) **CNF count is inversely proportional to the amount of dysferlin expressed in each line, except in KO-3 which have similar CNF counts to KO-1 samples. BLAJs recorded the highest CNF counts ~4-fold higher than the KO-1/BLAJ line. **(C)** Quantification of muscle tissue cross-sectional area revealed a significant reduction in BLAJ mice, whereas 40aKO lines are similar to WT mice. **(D) **Plot of frequency distribution of muscle fiber diameter in the six different lines. BLAJ mice predominately have smaller fibers whereas 40aKO line show a trend for slightly larger fibers or similarly sized fibers compared to WT. **(E) **Analysis of percentage variation in fiber diameter revealed significant increase fiber diameter variation in the KO-1/BLAJ line similar to BLAJ mice. The rest of the 40aKO lines show fiber diameter variation similar to WT. **(F)** Fat deposit quantification by oil-red O staining revealed no significant differences between genotypes. In all graphs, each dot represents an individual animal, n = 3-4/genotype. Data are expressed as mean ± SEM. Differences were tested by One-way ANOVA with Tukey’s multiple-comparisons test. *p<0.05, **p<0.01, ***p<0.001.

**Additional file 6.** **Fig. S6.** Gene ontology and KEGG pathway analysis of dysferlin 40aKO, WT and BLAJ mice. Functional enrichment analysis with functional annotation from UniProt’s Gene Ontology (GO) and KEGG pathway databases, was performed on proteomics datasets from *Dysf *40aKO lines expressing variable amounts of dysferlin protein (KO-1_low_, KO-2_mid_ and KO-3_high_), control WT and dysferlin-null BLAJ mice (all aged 18weeks). Data was acquired across two different experiments, the first set comprising KO-1, KO-2, WT and BLAJ, and the second set comprising KO-3, KO-2, WT and BLAJ, n = 4 samples/group. Pair-wise comparisons were conducted across the two different experiments to identify significantly altered GO and KEGG pathway terms as displayed in the graphs. For each pair-wise comparison (e.g BLAJ vs WT), an upward red arrow indicates upregulation of the term on the y-axis with respect to the first group i.e BLAJ in this example. Conversely, a blue downward arrow indicates downregulation of the term on the y-axis. If a pair-wise comparison has been omitted in a graph, it indicates no significant differences were identified between groups for a particular GO/KEGG pathway term. Lack of or reduced expression of dysferlin affected a number of biological, molecular processes and cellular components. Notably terms related to lipid metabolism, were significantly downregulated in BLAJ relative to WT, but normal in almost all KO lines.

**Additional file 7.** Supplementary Materials and Methods.

**Additional file 8.** **Supplementary Table 1.** PCR primers used to probe dysferlin Exon 40a locus.

**Additional file 9.** **Table 2.** List of Common targets in Expt 1 and Expt 2_BLAJ vs WT_and KO-2 vs BLAJ.

**Additional file 10.** **Table 3.** Dysferlin interacting partners-anova_posthoc.

**Additional file 11.** **Table 4.** Lipid metabolism_anova_posthoc.

**Additional file 12.** **Table 5.** Contractile and Structural proteins_anova_posthoc.

**Additional file 13.** **Table 6.** ECM proteins_anova_posthoc

**Additional file 14** **Table 7 ** Mitochondrial and oxidative proteins_anova_posthoc.

**Additional file 15.** Copy of unedited blots and gels.

**Additional file 16.** **Fig. S7.** Absence of endogenous minidysferlin_C72_ in dysferlin 40aKO lines. Endogenous dysferlin was immunoprecipitated from WT, 40aKO lines 1-to-3 and BLAJ muscle tissue lysates using N-terminal antibody Romeo and protein G–Sepharose beads. Dysferlin immobilised on sepharose beads was then digested with calpain-2 in the presence of 2 mM CaCl_2_. Dysferlin was detected by Western blot analysis with the C-terminal antibody Hamlet-1. Mini-dysferlin_C72_ is seen only in the calpain digested WT sample and not in any of the 40aKO or BLAJ samples.

**Additional file 17. Fig S8.** Absence of excess lipid staining in muscle sections of young 18wk dysferlin 40aKO lines, BLAJ and WT mice. Muscle sections from the psoas of 18-wk-old dysferlin 40aKO mice, WT and BLAJ mice (n = 4/group) were stained with oil-red O to assess presence of lipid deposits. Unlike aged BLAJ mice, all young mice including BLAJ mice, show little evidence of excess lipid deposits within the muscle tissue.

**Additional file 18.** **Fig. S9.** EM analysis showing lipid accumulation in muscle fibers from BLAJ mice but not dysferlin 40aKO-1_low_ line. EM analysis was conducted on frozen psoas muscle tissue from 12 month WT, dysferlin deficient BLAJ mice and hypomorphic dysferlin 40aKO-1_low_ lineOne mouse was examined per group. The 40aKO-1_low_ line, similar to the WT sample exhibited normal sarcomeric structure and absence of inter-myofibrillar lipid deposits, although occasional intramyofibrillar lipids (asterisk) were observed. The BLAJ muscle had extensive areas of lipid deposits which were predominantly intermyofiber adipocytes. There was evidence of intracellular lipid deposits in some but not all BLAJ muscle fibers (asterisks). Areas with disorganized sarcomeres (arrow) were also apparent in BLAJ muscle but not 40aKO-1_low_ or WT muscle.

## Data Availability

Data used in the current study has been provided in figures and supplementary material. Proteomics mass spectrometry datasets can be accessed from the PRIDE public repository using the following login details: WT-KO2-KO3-BLAJ-Oct2020:s Project Name: Minimal expression of dysferlin prevents development of muscular dystrophy in dysferlin exon 40a knockout mice, Project accession: PXD035393, Project https://doi.org/10.6019/PXD035393, Reviewer account details: Username: reviewer_pxd035393@ebi.ac.uk; Password: 5Q66e6Ye, WT-BLAJ-KO1-KO2-Jan2021: Project Name: Minimal expression of dysferlin prevents development of muscular dystrophy in dysferlin exon 40a knockout mice (second dataset), Project accession: PXD035918. Project https://doi.org/10.6019/PXD035918, Reviewer account details: Username: reviewer_pxd035918@ebi.ac.uk; Password: XFvCo4Dy.
