# Supplementary material for: Minimal expression of dysferlin prevents development of dysferlinopathy in dysferlin exon 40a knockout mice
Source: Acta Neuropathol Commun. 2023 Jan 18;11:15. doi: 10.1186/s40478-022-01473-x (PMC9847081; doi:10.1186/s40478-022-01473-x)
Supplement: Supplementary file 1 — Additional file 1. Fig. S1. Genetic characterisation of dysferlin exon 40a knockout mice following CRISPR/Cas-9 genome editing. (A) Microdeletion events in and around dysferlin exon 40a in the three dysferlin exon 40a knockout mice generated by CRISPR/Cas-9 gene editing. 40aKO-1 carries a 24 bp deletion preceding the splice acceptor. 40aKO-2 carries a 65 bp deletion spanning intron 40 and exon 40a. 40aKO-3 carries an in-frame 12 bp deletion within exon 40a. Sanger sequencing primers flanking exon 40a (red text) were used to determine the precise genomic variations induced by CRISPR/Cas-9 activity. (B) PCR screening of genomic DNA from WT and dysferlin exon 40a knockout mice using primers that flank exon 40a (red text). PCR product sizes are indicated on the gel (C) PCR amplification of cDNA derived from the skeletal muscle and the lung of a WT and exon 40aKO-1 homozygous mouse. Using an upstream cryptic acceptor splice site (black arrowhead in A), ectopic intron 40 sequence and exon 40a is more frequently spliced into 40aKO-1 Dysf transcripts relative to exon 40a inclusion into Dysf transcripts of WT mice. 40aKO-1 transcripts utilising the upstream cryptic acceptor (black arrowhead in A) encode a premature termination codon (PTC) and should thus be subject to nonsense-mediated decay (NMD). (D) PCR amplification of cDNA derived from the skeletal muscle of three exon 40aKO-2 mice and one WT mouse. The upstream cryptic acceptor (black arrowhead in A) is also utilised in 40aKO-2 mice to splice in ectopic intron 40 sequence and exon 40a into Dysf transcripts more frequently than exon 40a is spliced into Dysf transcripts of WT mice. These transcripts (upper band in 40aKO-2) encode a PTC and should thus be subject to NMD. (E) Dysferlin exon 40aKO-3 mice exclusively express Dysf transcripts lacking exon 40a. These transcripts are in-frame and predicted to encode full length dysferlin. In skeletal muscle, there is a minor Dysf transcript expressed that lacks exon 41. [file 40478_2022_1473_MOESM1_ESM.pdf]

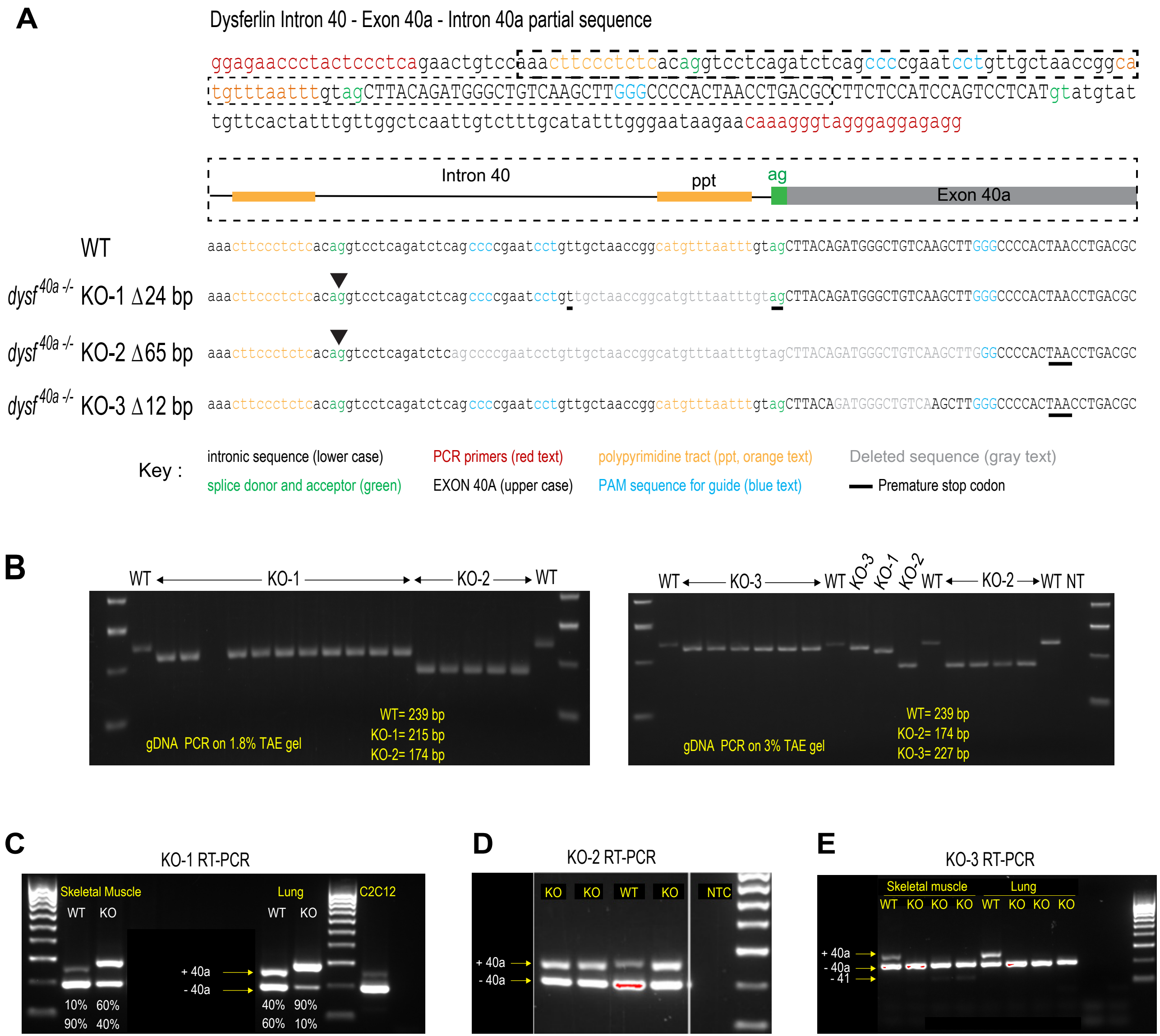

A
Dysferlin Intron 40 - Exon 40a - Intron 40a partial sequence
ggagaaccctactccctcagaactgtccaaacttccctctcacaggtcctcagatctcagccccgaatcctgttgctaaccggca
tgtttaatttgtagCTTACAGATGGGCTGTCAAGCTTGGGCCCCACTAACCTGACGCCTTCTCCATCCAGTCCTCATgtatgtat
tgttcactatttgttggctcaattgtctttgcatatttgggaataagaacaaagggtagggaggagagg
Intron 40
ppt
ag
Exon 40a
WT
aaacttccctctcacaggtcctcagatctcagccccgaatcctgttgctaaccggcatgtttaatttgtagCTTACAGATGGGCTGTCAAGCTTGGGCCCCACTAACCTGACGC
dysf 40a -/- KO-1 Δ24 bp
aaacttccctctcacaggtcctcagatctcagccccgaatcctgttgctaaccggcatgtttaatttgtagCTTACAGATGGGCTGTCAAGCTTGGGCCCCACTAACCTGACGC
dysf 40a -/- KO-2 Δ65 bp
aaacttccctctcacaggtcctcagatctcagccccgaatcctgttgctaaccggcatgtttaatttgtagCTTACAGATGGGCTGTCAAGCTTGGGCCCCACTAACCTGACGC
dysf 40a -/- KO-3 Δ12 bp
aaacttccctctcacaggtcctcagatctcagccccgaatcctgttgctaaccggcatgtttaatttgtagCTTACAGATGGGCTGTCAAGCTTGGGCCCCACTAACCTGACGC
Key :
intronic sequence (lower case)
PCR primers (red text)
polypyrimidine tract (ppt, orange text)
Deleted sequence (gray text)
splice donor and acceptor (green)
EXON 40A (upper case)
PAM sequence for guide (blue text)
Premature stop codon
B
WT KO-1 KO-2 WT
gDNA PCR on 1.8% TAE gel
WT= 239 bp
KO-1= 215 bp
KO-2= 174 bp
WT KO-3 WT KO-3 KO-1 KO-2 WT KO-2 WT NT
gDNA PCR on 3% TAE gel
WT= 239 bp
KO-2= 174 bp
KO-3= 227 bp
C
KO-1 RT-PCR
Skeletal Muscle
WT KO
Lung
WT KO
C2C12
+ 40a
- 40a
10% 60%
90% 40%
40% 90%
60% 10%
D
KO-2 RT-PCR
KO KO WT KO NTC
+ 40a
- 40a
E
KO-3 RT-PCR
Skeletal muscle
Lung
WT KO KO KO WT KO KO KO
+ 40a
- 40a
- 41
