# Supplementary figures and images for: Minimal expression of dysferlin prevents development of dysferlinopathy in dysferlin exon 40a knockout mice
Source: Acta Neuropathol Commun. 2023 Jan 18;11:15. doi: 10.1186/s40478-022-01473-x (PMC9847081; doi:10.1186/s40478-022-01473-x)

**A**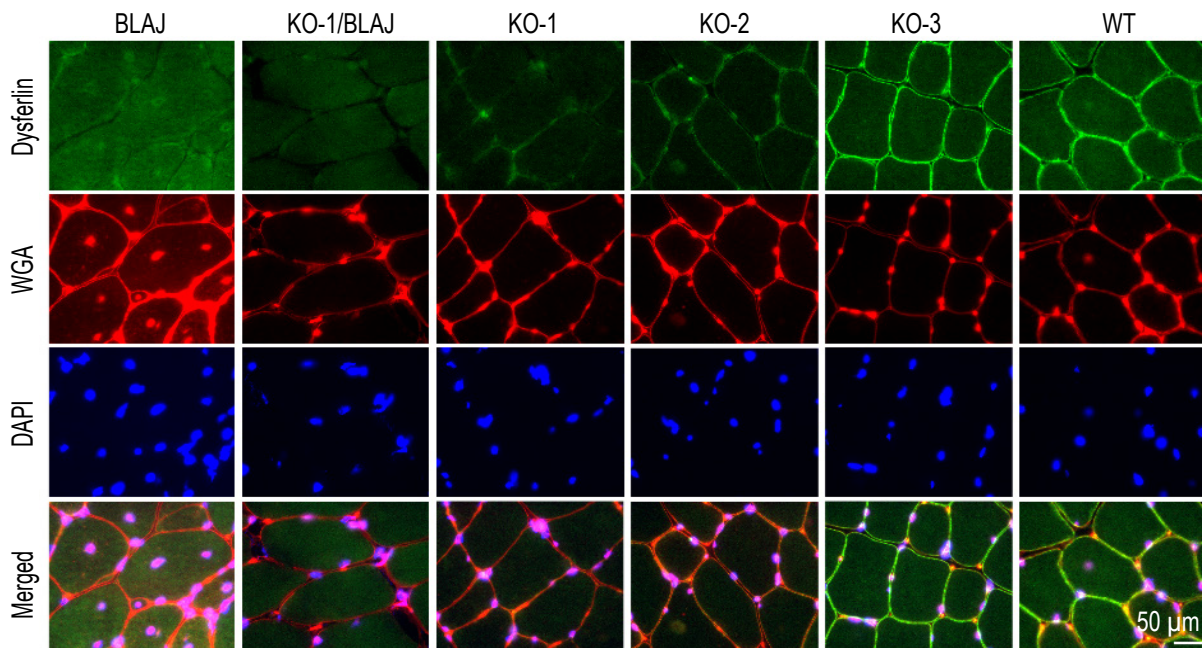**B**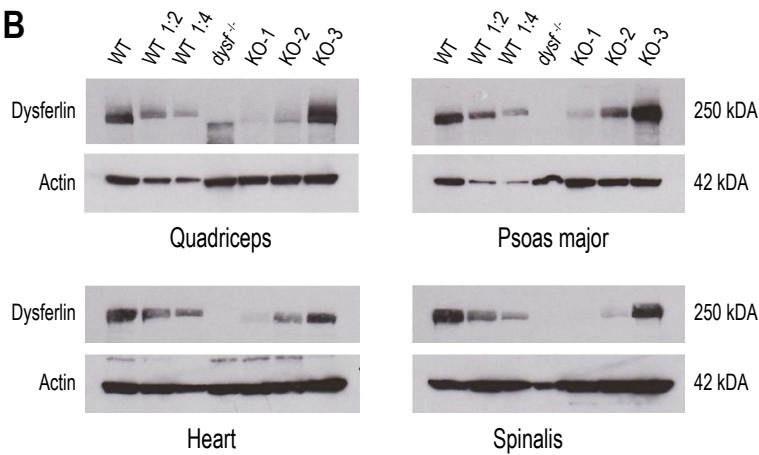**C**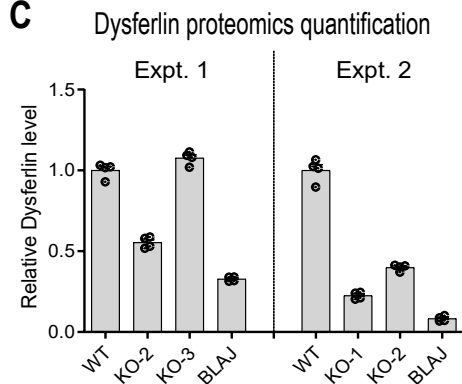

Supplement: Supplementary file 2 — Additional file 2. Fig. S2. Localisation and quantification of dysferlin protein expression in dysferlin exon 40a knockout mice generated by CRISPR/Cas-9 genome editing. (A) Immunofluorescent staining for dysferlin using NCL Hamlet-1 on quadriceps muscle sections from 12-month-old WT, dysferlin 40aKO and BLAJ mice. Dysferlin is predominantly expressed at the sarcolemma but is reduced to varying degrees in the 40aKO lines compared to WT. Images representative of n = 3/group. (B) Western blot of protein extracted from quadriceps, psoas major, heart and spinalis muscles indicated that dysferlin protein levels in Dysf 40aKO lines (abbreviated KO-1-to-3) relative to WT are as follows: ~10-20% in KO-1, ~50% in KO-2 and ~90-100% in KO-3. (C) Proteomic analysis of dysferlin protein levels in quadriceps muscles of WT, BLAJ and exon 40aKO mice (n = 4/group), revealed similar trends to semiquantitative estimates by Western blot (B), except for higher dysferlin estimates in BLAJ mice (~10-25% of WT), particularly in experiment 1. Graphed data is represented as mean ± SEM. [file 40478_2022_1473_MOESM2_ESM.pdf]

# Quadriceps

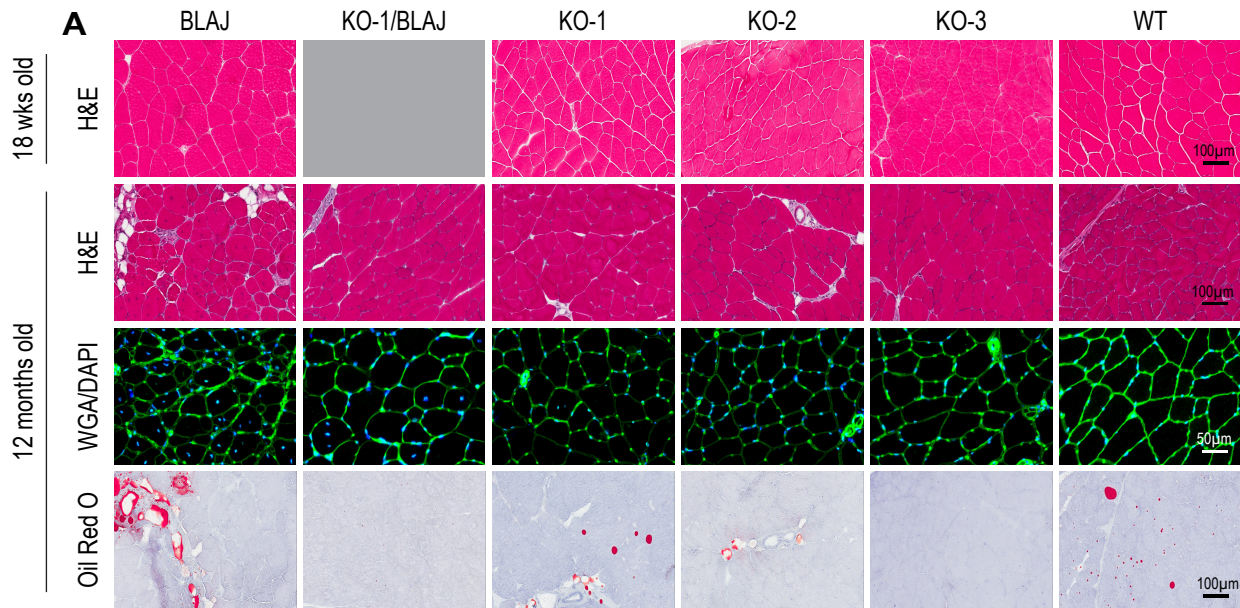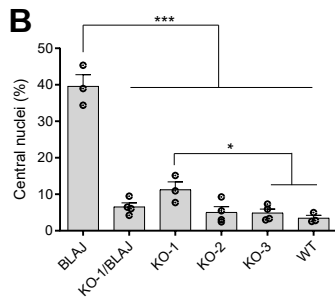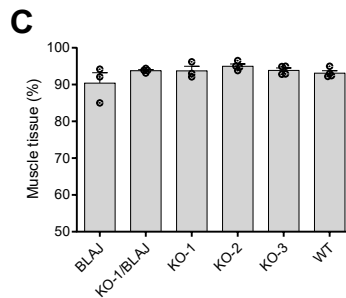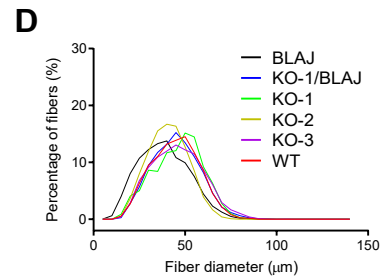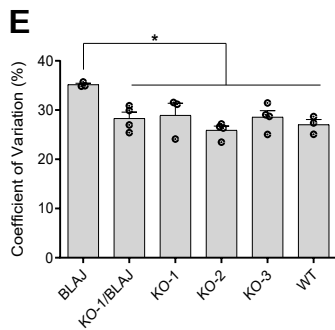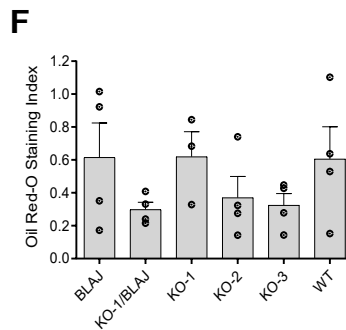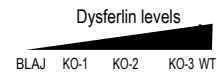

Supplement: Supplementary file 3 — Additional file 3. Fig. S3. Histopathological analysis of quadriceps muscles of 40aKO mice. (A) Haematoxylin and eosin stain of young 18 wk old mice, including BLAJ mice, shows lack of overt dystrophic features (centralised nuclei, fibro-fatty deposits) in any of these mice. The H&E image of 18wk old KO-1/BLAJ has intentionally been omitted because we did not have samples from this line at this age. Compared to aged (~12 months) BLAJ mice, age-matched 40aKO mice do not display signs of overt dystrophy. (B) Quantification of central nucleated fibers (CNF) from WGA/DAPI stained images shows significantly reduced CNF counts in 40aKO mice compared to BLAJs. CNF counts of KO-2 and KO-3 are similar to that of WT. KO-1 and KO-1/BLAJ have slightly elevated CNF counts relative to WT. There is no significant difference between 40aKO lines, WT and BLAJ muscle tissue cross-sectional area (C), fiber diameter size frequency distribution (D) and oil-red O staining index (lipid content, F, however, there is significant fiber size variation in BLAJ mice (E). In all graphs, each dot represents an individual animal, n = 3-4/genotype, data is represented as mean ±SEM, differences between groups were assessed by One-way ANOVA followed by Tukey’s multiple comparison test. [file 40478_2022_1473_MOESM3_ESM.pdf]

# Glutues

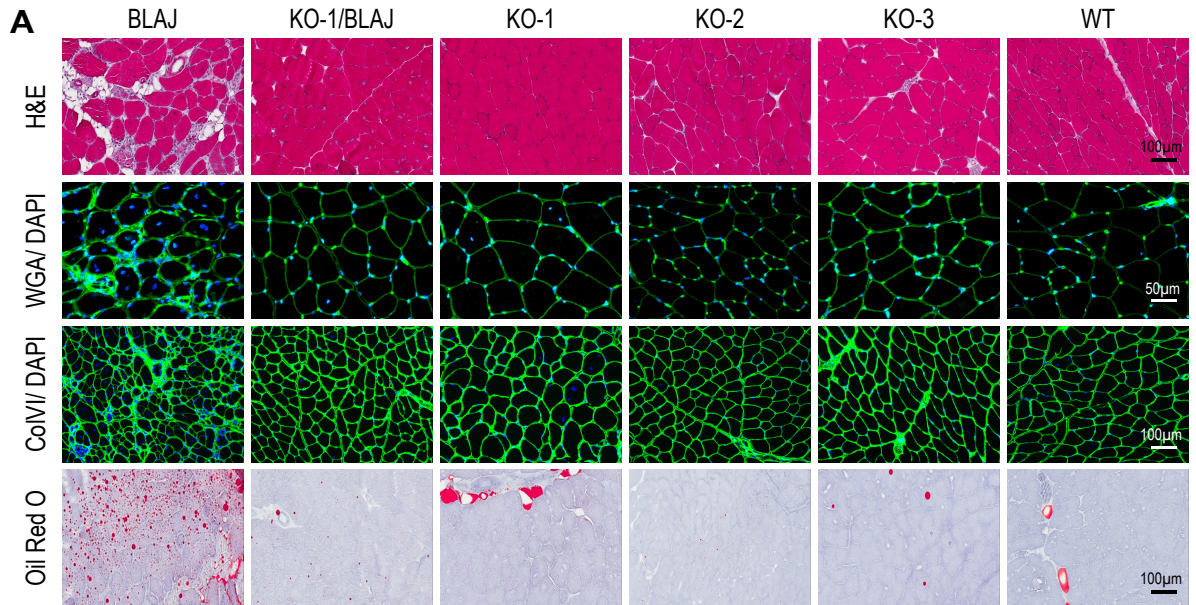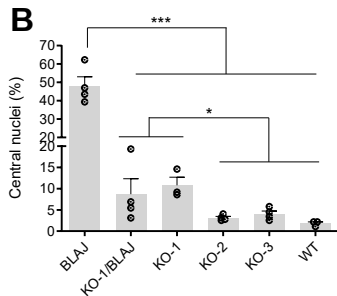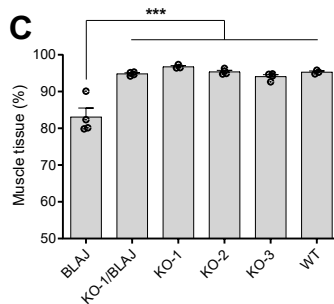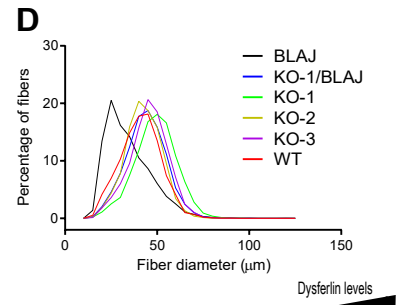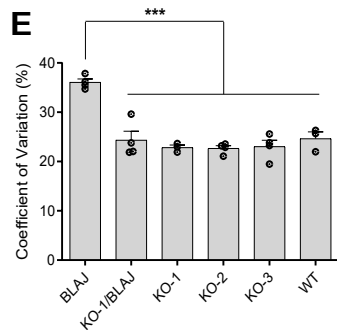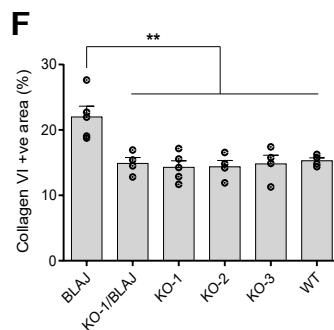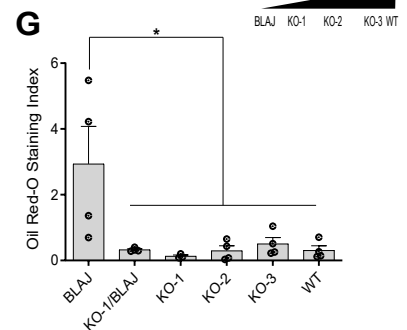

Supplement: Supplementary file 4 — Additional file 4. Fig. S4. Histopathological analysis of gluteus muscles from aged 40aKO mice. (A) Representative images of gluteus muscle sections from 12-month-old 40aKO mice, WT and BLAJ stained with H&E (morphology), WGA/DAPI (CNF counts), Col VI/DAPI (fibrosis) and oil-red O (fat deposition) evaluation. 40aKO mice are histologically similar to WT, lacking overt dystrophic features seen in BLAJ mice. (B) CNF count is inversely proportional to the amount of dysferlin expressed. KO-2 and KO-3 are not significantly different from WT. KO-1 and KO-1/BLAJ CNF counts are slightly but significantly elevated relative to KO-2 and KO-3. BLAJs have the highest CNF count, at least 5-fold more than KO-1. Quantitative analysis of muscle tissue cross-sectional area (C), fiber diameter frequency distribution (D) and size variation (E), collagen content (F) and fat deposition (G), revealed no significant differences between 40aKO lines and WT, in contrast to BLAJs which showed significant alterations in all these parameters. In all graphs, each dot represents an individual animal, n = 3-4 in B-E and G, n = 4-5 in F. Data are expressed as mean ± SEM. Differences were tested by One-way ANOVA with Tukey’s multiple-comparisons test. *p<0.05, **p<0.01, ***p<0.001. [file 40478_2022_1473_MOESM4_ESM.pdf]

# Spinalis

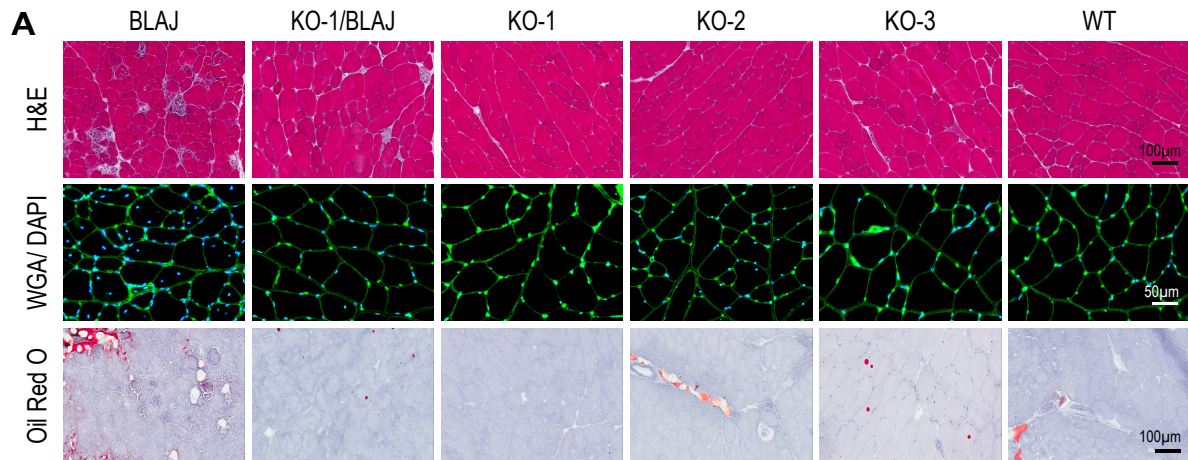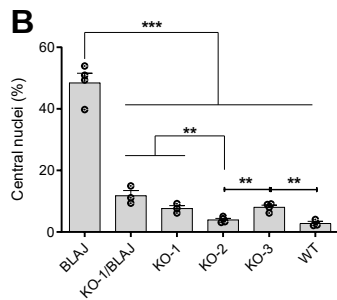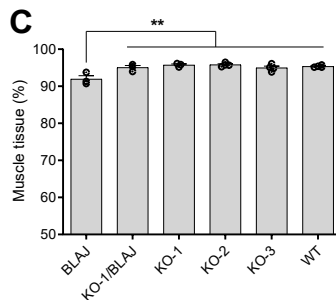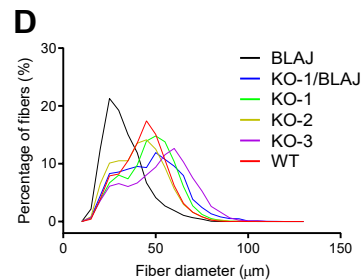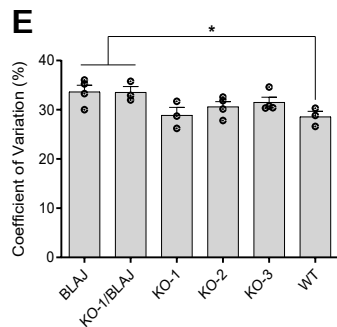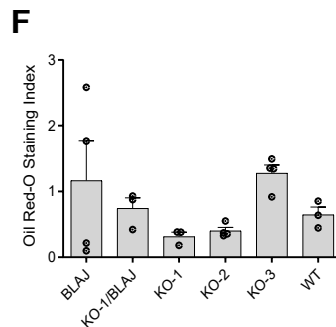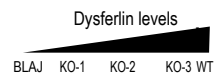

Supplement: Supplementary file 5 — Additional file 5. Fig. S5. Histopathological analysis of spinalis muscles from aged 40aKO mice. (A) Muscle sections from spinalis of 12-month-old dysferlin 40aKO mice, WT and BLAJ mice were stained with H&E, WGA/DAPI and oil-red O to assess their morphology, presence of centrally nucleated fibers (CNF) and fatty deposition respectively. (B) CNF count is inversely proportional to the amount of dysferlin expressed in each line, except in KO-3 which have similar CNF counts to KO-1 samples. BLAJs recorded the highest CNF counts ~4-fold higher than the KO-1/BLAJ line. (C) Quantification of muscle tissue cross-sectional area revealed a significant reduction in BLAJ mice, whereas 40aKO lines are similar to WT mice. (D) Plot of frequency distribution of muscle fiber diameter in the six different lines. BLAJ mice predominately have smaller fibers whereas 40aKO line show a trend for slightly larger fibers or similarly sized fibers compared to WT. (E) Analysis of percentage variation in fiber diameter revealed significant increase fiber diameter variation in the KO-1/BLAJ line similar to BLAJ mice. The rest of the 40aKO lines show fiber diameter variation similar to WT. (F) Fat deposit quantification by oil-red O staining revealed no significant differences between genotypes. In all graphs, each dot represents an individual animal, n = 3-4/genotype. Data are expressed as mean ± SEM. Differences were tested by One-way ANOVA with Tukey’s multiple-comparisons test. *p<0.05, **p<0.01, ***p<0.001. [file 40478_2022_1473_MOESM5_ESM.pdf]

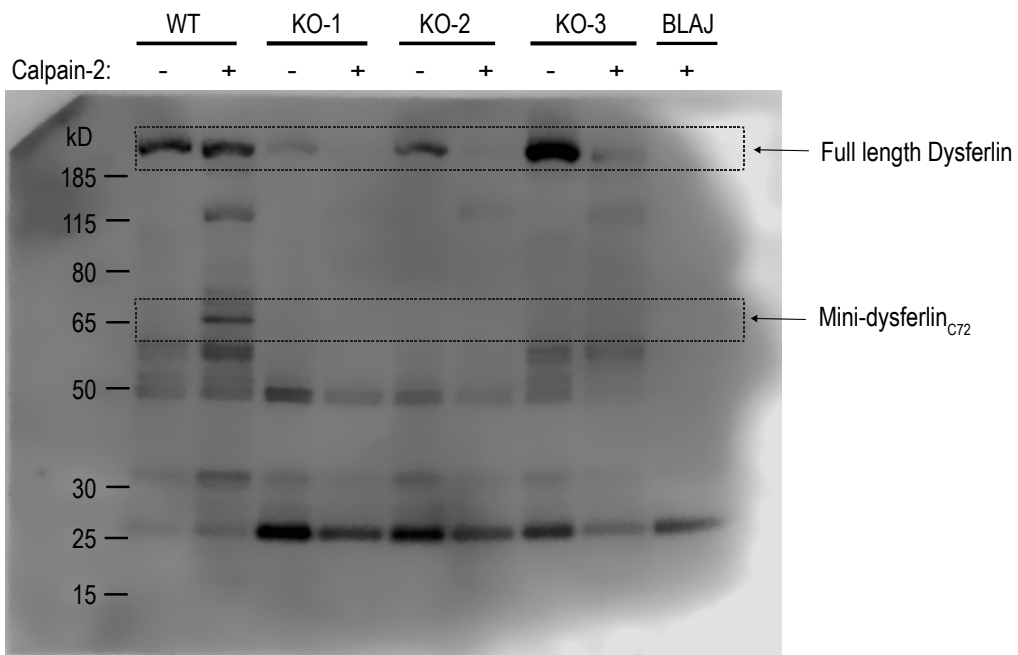

Supplement: Supplementary file 16 — Additional file 16. Fig. S7. Absence of endogenous minidysferlinC72 in dysferlin 40aKO lines. Endogenous dysferlin was immunoprecipitated from WT, 40aKO lines 1-to-3 and BLAJ muscle tissue lysates using N-terminal antibody Romeo and protein G–Sepharose beads. Dysferlin immobilised on sepharose beads was then digested with calpain-2 in the presence of 2 mM CaCl2. Dysferlin was detected by Western blot analysis with the C-terminal antibody Hamlet-1. Mini-dysferlinC72 is seen only in the calpain digested WT sample and not in any of the 40aKO or BLAJ samples. [file 40478_2022_1473_MOESM16_ESM.pdf]

18 wk old Psoas

BLAJ

KO-1

KO-2

KO-3

WT

#1

#2

#3

#4

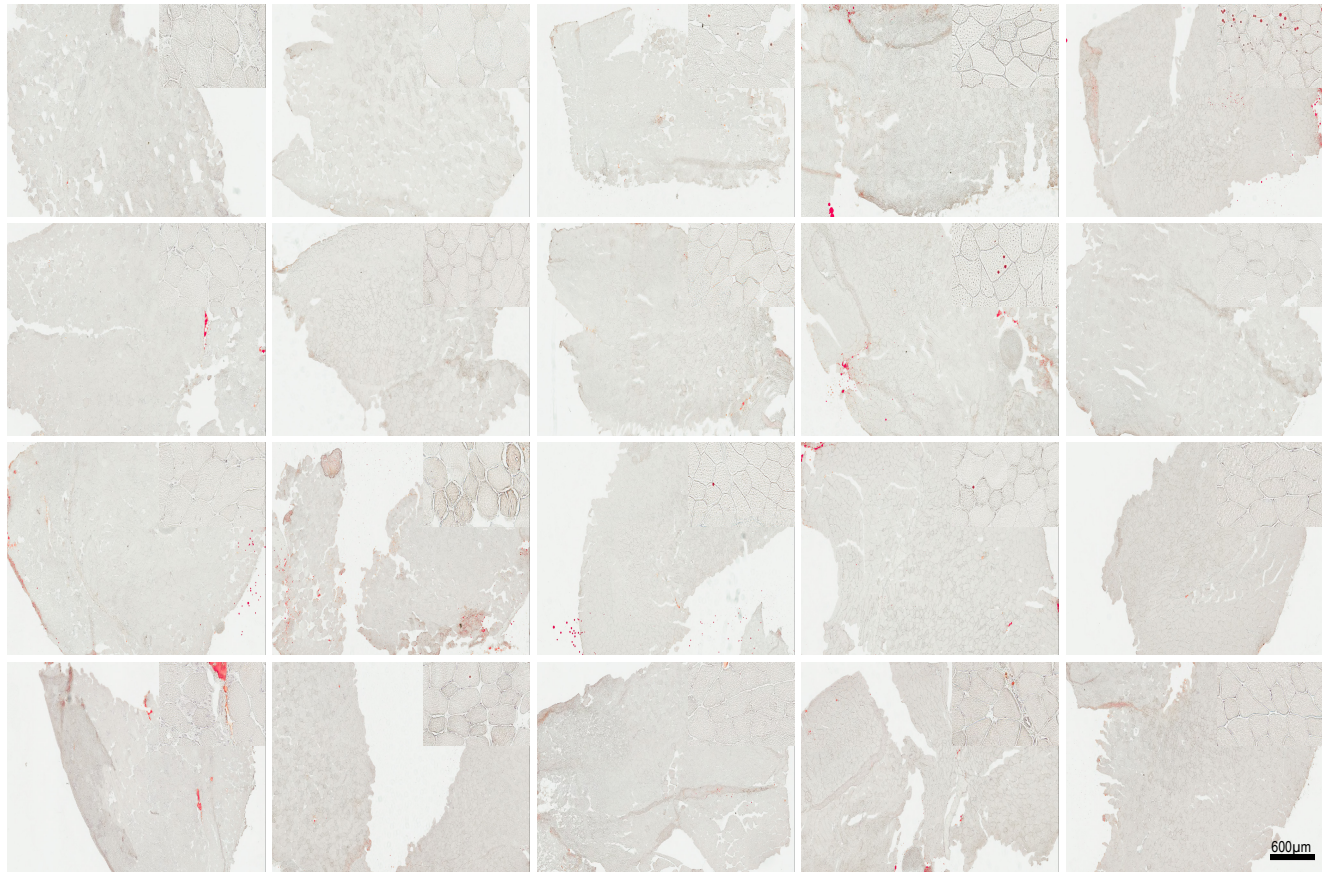

Supplement: Supplementary file 17 — Additional file 17. Fig S8. Absence of excess lipid staining in muscle sections of young 18wk dysferlin 40aKO lines, BLAJ and WT mice. Muscle sections from the psoas of 18-wk-old dysferlin 40aKO mice, WT and BLAJ mice (n = 4/group) were stained with oil-red O to assess presence of lipid deposits. Unlike aged BLAJ mice, all young mice including BLAJ mice, show little evidence of excess lipid deposits within the muscle tissue. [file 40478_2022_1473_MOESM17_ESM.pdf]

WT

KO-1

BLAJ

x500

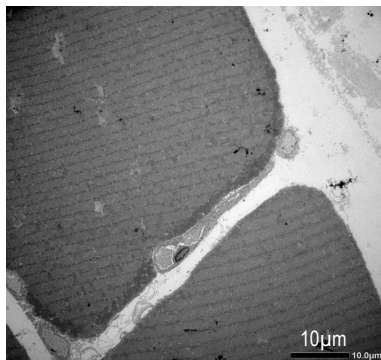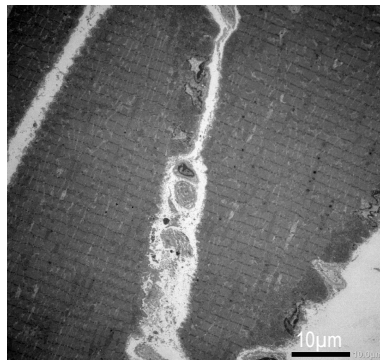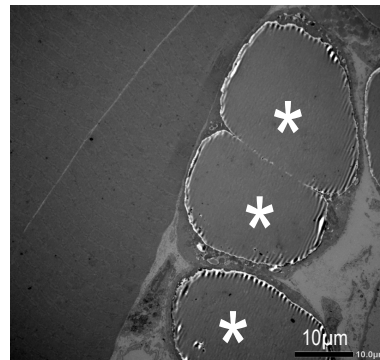

x2000

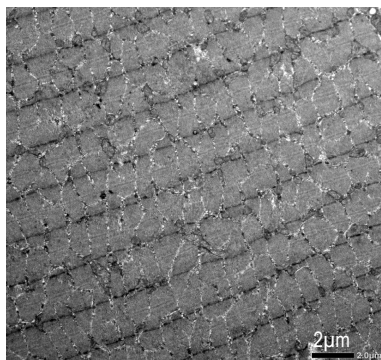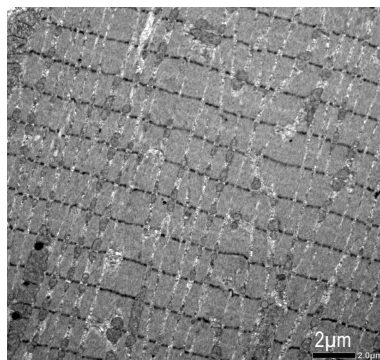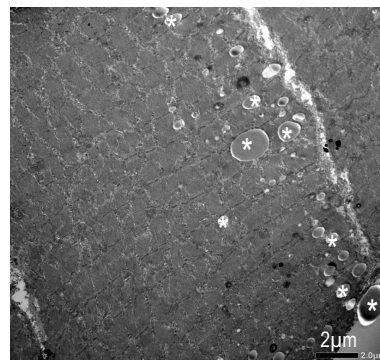

x10000

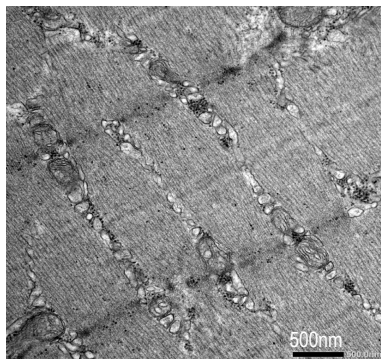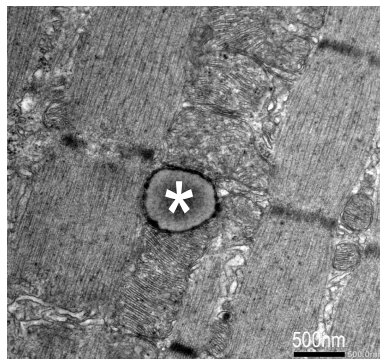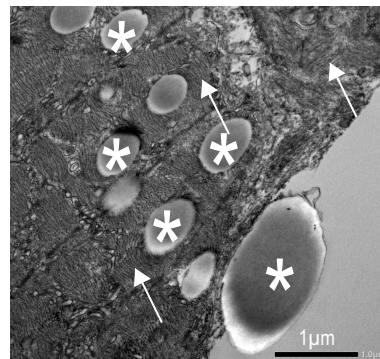

Supplement: Supplementary file 18 — Additional file 18. Fig. S9. EM analysis showing lipid accumulation in muscle fibers from BLAJ mice but not dysferlin 40aKO-1low line. EM analysis was conducted on frozen psoas muscle tissue from 12 month WT, dysferlin deficient BLAJ mice and hypomorphic dysferlin 40aKO-1low lineOne mouse was examined per group. The 40aKO-1low line, similar to the WT sample exhibited normal sarcomeric structure and absence of inter-myofibrillar lipid deposits, although occasional intramyofibrillar lipids (asterisk) were observed. The BLAJ muscle had extensive areas of lipid deposits which were predominantly intermyofiber adipocytes. There was evidence of intracellular lipid deposits in some but not all BLAJ muscle fibers (asterisks). Areas with disorganized sarcomeres (arrow) were also apparent in BLAJ muscle but not 40aKO-1low or WT muscle. [file 40478_2022_1473_MOESM18_ESM.pdf]
