# Supplementary material for: Minimal expression of dysferlin prevents development of dysferlinopathy in dysferlin exon 40a knockout mice
Source: Acta Neuropathol Commun. 2023 Jan 18;11:15. doi: 10.1186/s40478-022-01473-x (PMC9847081; doi:10.1186/s40478-022-01473-x)
Supplement: Supplementary file 6 — Additional file 6. Fig. S6. Gene ontology and KEGG pathway analysis of dysferlin 40aKO, WT and BLAJ mice. Functional enrichment analysis with functional annotation from UniProt’s Gene Ontology (GO) and KEGG pathway databases, was performed on proteomics datasets from Dysf 40aKO lines expressing variable amounts of dysferlin protein (KO-1low, KO-2mid and KO-3high), control WT and dysferlin-null BLAJ mice (all aged 18weeks). Data was acquired across two different experiments, the first set comprising KO-1, KO-2, WT and BLAJ, and the second set comprising KO-3, KO-2, WT and BLAJ, n = 4 samples/group. Pair-wise comparisons were conducted across the two different experiments to identify significantly altered GO and KEGG pathway terms as displayed in the graphs. For each pair-wise comparison (e.g BLAJ vs WT), an upward red arrow indicates upregulation of the term on the y-axis with respect to the first group i.e BLAJ in this example. Conversely, a blue downward arrow indicates downregulation of the term on the y-axis. If a pair-wise comparison has been omitted in a graph, it indicates no significant differences were identified between groups for a particular GO/KEGG pathway term. Lack of or reduced expression of dysferlin affected a number of biological, molecular processes and cellular components. Notably terms related to lipid metabolism, were significantly downregulated in BLAJ relative to WT, but normal in almost all KO lines. [file 40478_2022_1473_MOESM6_ESM.pdf]

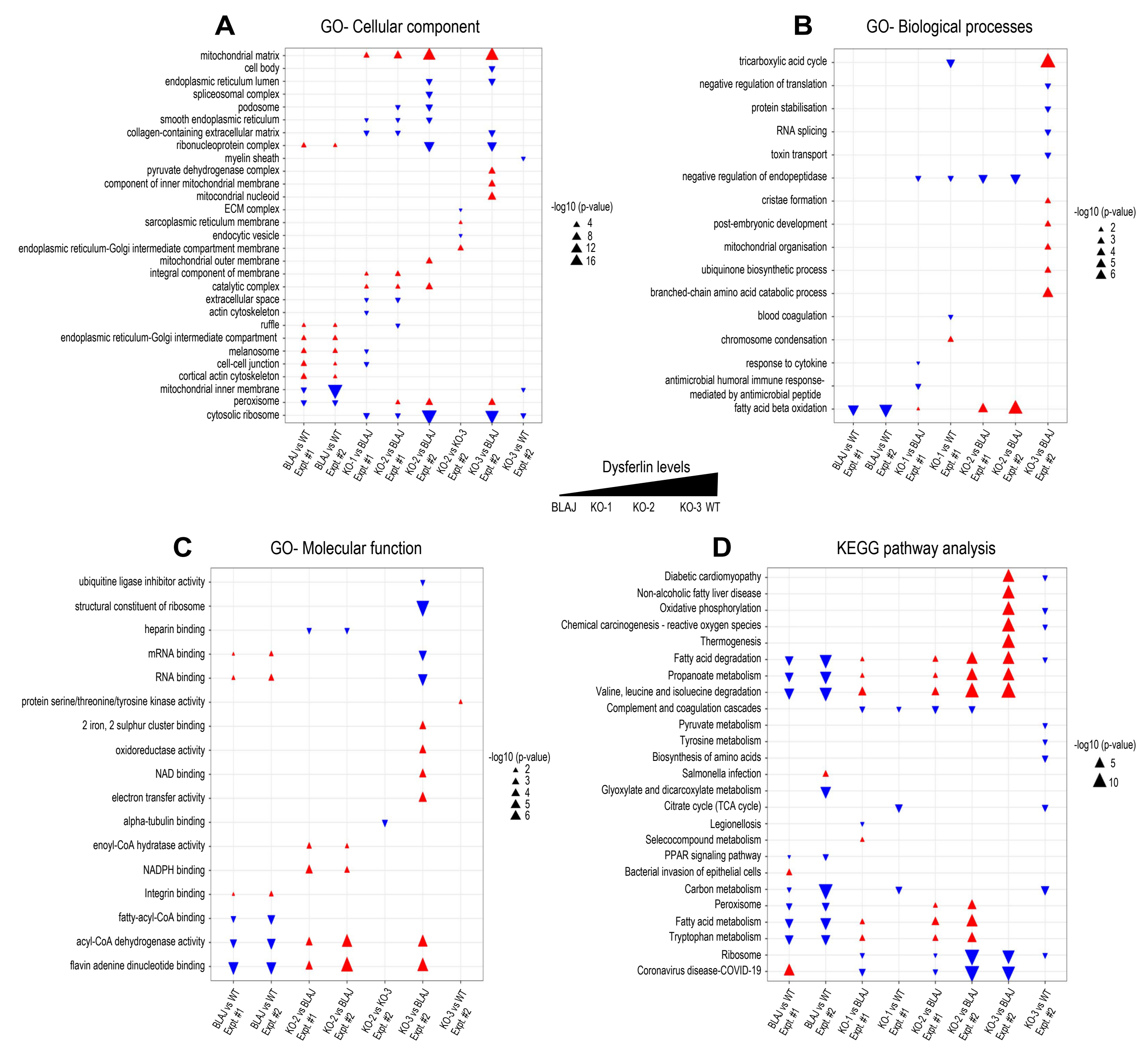
A
GO- Cellular component
mitochondrial matrix
cell body
endoplasmic reticulum lumen
spliceosomal complex
podosome
smooth endoplasmic reticulum
collagen-containing extracellular matrix
ribonucleoprotein complex
myelin sheath
pyruvate dehydrogenase complex
component of inner mitochondrial membrane
mitochondrial nucleoid
ECM complex
sarcoplasmic reticulum membrane
endocytic vesicle
endoplasmic reticulum-Golgi intermediate compartment membrane
mitochondrial outer membrane
integral component of membrane
catalytic complex
extracellular space
actin cytoskeleton
ruffle
endoplasmic reticulum-Golgi intermediate compartment
melanosome
cell-cell junction
cortical actin cytoskeleton
mitochondrial inner membrane
peroxisome
cytosolic ribosome
BLAJ vs WT Expt. #1
BLAJ vs WT Expt. #2
KO-1 vs BLAJ Expt. #1
KO-2 vs BLAJ Expt. #1
KO-2 vs BLAJ Expt. #2
KO-2 vs KO-3 Expt. #2
KO-3 vs BLAJ Expt. #2
KO-3 vs WT Expt. #2
-log10 (p-value)
4
8
12
16
B
GO- Biological processes
tricarboxylic acid cycle
negative regulation of translation
protein stabilisation
RNA splicing
toxin transport
negative regulation of endopeptidase
cristae formation
post-embryonic development
mitochondrial organisation
ubiquinone biosynthetic process
branched-chain amino acid catabolic process
blood coagulation
chromosome condensation
response to cytokine
antimicrobial humoral immune response-mediated by antimicrobial peptide
fatty acid beta oxidation
BLAJ vs WT Expt. #1
BLAJ vs WT Expt. #2
KO-1 vs BLAJ Expt. #1
KO-1 vs WT Expt. #1
KO-2 vs BLAJ Expt. #1
KO-2 vs BLAJ Expt. #2
KO-3 vs BLAJ Expt. #2
-log10 (p-value)
2
3
4
5
6
Dysferlin levels
BLAJ
KO-1
KO-2
KO-3
WT
C
GO- Molecular function
ubiquitine ligase inhibitor activity
structural constituent of ribosome
heparin binding
mRNA binding
RNA binding
protein serine/threonine/tyrosine kinase activity
2 iron, 2 sulphur cluster binding
oxidoreductase activity
NAD binding
electron transfer activity
alpha-tubulin binding
enoyl-CoA hydratase activity
NADPH binding
Integrin binding
fatty-acyl-CoA binding
acyl-CoA dehydrogenase activity
flavin adenine dinucleotide binding
BLAJ vs WT Expt. #1
BLAJ vs WT Expt. #2
KO-2 vs BLAJ Expt. #1
KO-2 vs BLAJ Expt. #2
KO-2 vs KO-3 Expt. #2
KO-3 vs BLAJ Expt. #2
KO-3 vs WT Expt. #2
-log10 (p-value)
2
3
4
5
6
D
KEGG pathway analysis
Diabetic cardiomyopathy
Non-alcoholic fatty liver disease
Oxidative phosphorylation
Chemical carcinogenesis - reactive oxygen species
Thermogenesis
Fatty acid degradation
Propanoate metabolism
Valine, leucine and isoleucine degradation
Complement and coagulation cascades
Pyruvate metabolism
Tyrosine metabolism
Biosynthesis of amino acids
Salmonella infection
Glyoxylate and dicarcoxylate metabolism
Citrate cycle (TCA cycle)
Legionellosis
Selecocompound metabolism
PPAR signaling pathway
Bacterial invasion of epithelial cells
Carbon metabolism
Peroxisome
Fatty acid metabolism
Tryptophan metabolism
Ribosome
Coronavirus disease-COVID-19
BLAJ vs WT Expt. #1
BLAJ vs WT Expt. #2
KO-1 vs BLAJ Expt. #1
KO-1 vs WT Expt. #1
KO-2 vs BLAJ Expt. #1
KO-2 vs BLAJ Expt. #2
KO-3 vs BLAJ Expt. #2
KO-3 vs WT Expt. #2
-log10 (p-value)
5
10
