# Supplementary material for: Minimal expression of dysferlin prevents development of dysferlinopathy in dysferlin exon 40a knockout mice
Source: Acta Neuropathol Commun. 2023 Jan 18;11:15. doi: 10.1186/s40478-022-01473-x (PMC9847081; doi:10.1186/s40478-022-01473-x)
Supplement: Supplementary file 7 — Additional file 7. Supplementary Materials and Methods. [file 40478_2022_1473_MOESM7_ESM.pdf]

**Generation and characterisation of exon40a mouse lines.**

The murine genomic locus of dysferlin encompassing exon 40a was submitted to the online CRISPR guide design tool (http://crispr.mit.edu/) to determine the location of CRISPR/Cas9 guide sequences that could disrupt expression of exon 40a. To minimise off-target effects we chose to use paired guides along with a nickase (D10A single stranded cutter) version of Cas9. Guides were synthesised by SIGMA Australia and cloned into the pSpCas9n(BB)-2A-Puro (PX462) V1.0 vector obtained from ADDGENE (plasmid #48141, a kind gift from Feng Zhang) as previously published (Ran et al, Nature Protocols 2013).

Pronuclear C57BL/6 embryos were injected with in-vitro transcribed mRNA. For Cas9n (D10A) this was synthesized with the mMESSAGE mMACHINE™ T7 ULTRA Transcription Kit (Invitrogen) using 1ug gel-purified Cas9 D10A plasmid template (linearised using SmaI and MfeI) and a 6-hour synthesis time, followed by purification using the RNeasy Mini Kit (Qiagen). For the guides we used the MEGAshortscript™ T7 Transcription Kit (Invitrogen), PCR template (See Supplementary Table 1) with an overnight synthesis and subsequent clean-up using the ZYMO RESEARCH R1015 RNA Clean & Concentrator kit.

For pronuclear microinjection we prepared an injection mixture comprising two guides, guides (#2 & #9) at a final concentration of 50 ng/ul each and Cas9n at 100 ng/ul diluted in water. Embryos were injected in M2 medium (Sigma) containing cytochalasin B at 10 ug/ml to improve embryo survival (Hu et al, Zygote 2012). Injected embryos were incubated at 37$^{o}$C, 5% $CO_2$ until 2-cell stage and then implanted into pseudopregnant foster mothers (ARC Swiss outbred mice) at day 0.5 post-plug. Resulting pups were screened by PCR of genomic DNA across the exon 40a locus and non-WT sized PCR bands were sequenced.

Three potential founder pups that were identified by PCR with different sized deletions around and within exon40a were subsequently bred back to WT C57BL/6. All three founders

transmitted the mutant allele to offspring and the heterozygous offspring were intercrossed to generate homozygous animals for each line. Mendelian ratios were observed in the F2 and subsequent generations indicating no survival benefit or deficit associated with any of the deletions.

We also bred the 40aKO-$1_{low}$ line against BLAJ with the rationale that we may achieve a further reduction in dysferlin protein expression in these F1 mice compared to the 40aKO-1 parental line. Unfortunately, our western blot was unable to demonstrate if this held true as we were working at the lower limits of sensitivity of this assay and the antibodies we had available. As we had generated these animals we continued to age them and included them in our histopathology studies, but did not pursue them for the other arms of this project.

Line 40aKO-1 carries a 24-bp deletion of intronic sequence just upstream of the essential ag splice acceptor of exon 40a (intron 40) (Supp Fig 1A, 1B). Line 40aKO-2 carries a 65bp deletion comprising both 41bp of upstream intronic sequence as well as 24bp of exon 40a (Supp Fig 1A, 1B). Line 40aKO-3 carries a 12 bp deletion within exon 40a (Supp Fig 1A, 1B).

To determine the effect of these various deletions on expression of exon 40a we extracted mRNA from skeletal muscle and other tissues from homozygous animals from each line. We performed RT-PCR using primers anchored in the canonical exons upstream and downstream of exon 40a. (Supp Fig 1C, 1D, 1E).

Line exon 40AKO-1 demonstrated a significant skewing towards greater incorporation of exon 40a sequence than WT in all tissues examined (Supp Fig 1C). The transcripts incorporating the exon 40a sequence showed intron retention originating from a cryptic acceptor situated 55bp upstream of exon 40a, and presence of a premature termination codon (PTC) involving the essential (ag) splice acceptor of exon 40a. These transcripts were predicted to undergo nonsense-mediated decay (NMD). Any transcripts escaping NMD would result in dysferlin

protein carrying 9 missense amino acids and lacking 641 amino acids from the C-terminus, including the calpain cleavage site in exon 40a, the last two C2 domains, and the transmembrane domain.

Line exon 40AKO-2 demonstrated a skewing towards greater incorporation of exon 40a sequence than occurs in WT but not as profoundly as seen in line exon 40AKO-1 (Supp Fig 1D). The transcripts incorporating exon 40a sequence showed intron retention commencing from the same upstream cryptic acceptor as line 40aKO-1, and the presence of a PTC within the remaining exon 40a sequence. These transcripts were predicted to undergo nonsense-mediated decay (NMD). Any transcripts escaping NMD would result in dysferlin protein carrying 7 missense amino acids, disrupting the calpain cleavage site in exon 40a, and lacking at least 617 amino acids from the C-terminus, including the last two C2 domains, and the transmembrane domain.

Line exon 40AKO-$3_{high}$ did not show any detectable incorporation of exon 40a in all tissues examined. As well as skipping of exon 40a alone, we detected a proportion of transcripts with double skipping of exons 40 and 40a (seen as the faint band beneath the -40a dominant band in Supp. Fig 1E), resulting in a frame shift and rendering these transcripts subject to NMD. We also detected a minor pool of transcripts with double skipping of exons 40a and 41 (data not shown) which would result in loss of 162 bases and these transcripts would remain in frame.

To further understand these alterations to the splicing of exon 40a, we manually entered the murine dysferlin genomic sequence around exon 40a into a number of splicing prediction algorithms (including http://www.fruitfly.org/seq_tools/splice.html and www.lbgi.fr/spliceator/ (Reece et al, J Comp Biol 1997, Scalzitti et al, BMC Bioinformatics 2021). We note that while not all of the available algorithms are optimised for murine splicing,

due to the high conservation of splicing proteins and mechanisms we consider that predictions will likely hold for murine sequence as well as human.

In general the algorithms either did not recognise the canonical murine exon 40a splice acceptor, or recognised it but scored it as a very weak/poor acceptor sequence. This may be a contributing factor in the relatively low level of incorporation of this transcript in the normal dysferlin transcript pool in multiple tissues (Supp Fig 1C, 1D,1E). All algorithms did recognise the cryptic acceptor that is activated in lines 40KO-1 and 40AKO-2 and scored it very highly. This likely explains the much greater than usual incorporation of exon 40a sequence into the transcript pool in both these lines when the native acceptor is disrupted and the cryptic splice acceptor is activated.

We used RESCUE-ESE (Fairbrother et al, Nucleic Acids Research 2004) to examine the 12bp deleted sequence in line 40AKO-3 and found it contained some of the highest scoring exonic splice enhancer (ESE) motifs occurring within exon 40a. We surmised that the poor acceptor strength of exon 40a contributes to the alternative splicing of this exon, and that the ESE sequences that occur within exon 40a are essential for exon recognition by the murine splicing machinery. When the ESE's are disrupted, as in the case of line 40AKO-3, then exon 40a is rendered effectively invisible to the splice machinery and is not ever incorporated into the dysferlin transcript pool.

In line 40AKO-1, the high scoring ESEs in the first part of exon 40a remain, and along with the strong cryptic splice acceptor favour incorporation of the exon 40a sequence (including the retained intronic sequence) into the dysferlin transcript pool at much higher frequency than usual. The predominance of these transcripts that are predicted to be lost to NMD likely explains the significant reduction of dysferlin protein expression in this line, estimated to be at around 10-20% of WT levels (Supp Fig 1F, 1G, 1H).

In line 40aKO-$2_{mid}$ deletion of close to half of the exon 40a sequence removes the high scoring ESEs and thus tempers the incorporation of exon 40a sequence compared to line 40AKO-1, although it still occurs at much higher than normal levels due to use of the strong cryptic splice acceptor. The subsequent loss of these transcripts to NMD most likely explains the reduction in dysferlin protein expression in this line, which is estimated at around half of WT dysferlin levels (Supp Fig 1F, 1G, 1H).

We suspect the slight reduction in dysferlin protein expression in line 40aKO-$3_{high}$ (estimated to be at around 90-100% of WT levels) may be due to the presence of dysferlin transcripts lacking both exons 40 and 40a as this splicing outcome disrupts frame, creating a PTC which is predicted to target the transcripts for NMD. If translated, these transcripts would generate a dysferlin molecule carrying 5 missense amino acids, disrupting the fifth C2 domain (C2E) and then missing 642 amino acids from the C-terminus, including the final two C2 domains and the transmembrane domain.

The 40aKO-3 transcripts lacking exons 40a and 41 maintain frame and are predicted to generate a dysferlin isoform missing 33 amino acids from exon 41. Exon 41 carries a PEST sequence (sequences rich in proline (P), glutamic acid (E), serine (S) and threonine (T)) that may play a role in protein stability/protein degradation; however whether deletion of this sequence stabilises or destabilises dysferlin is unknown.

**References:**

Genome engineering using the CRISPR-Cas9 system. Ran FA, Hsu PD, Wright J, Agarwala V, Scott DA, Zhang F. *Nat Protoc. 2013 Nov:8(11):2281-308*. doi: 10.1038/nprot.2013.143

Hu LL, Shen XH, Zheng Z, Wang ZD, Liu ZH, Jin LH, Lei L. Cytochalasin B treatment of mouse oocytes during intracytoplasmic sperm injection (ICSI) increases embryo survival without impairment of development. Zygote. 2012 Nov;20(4):361-9. doi: 10.1017/S0967199411000438

Reese MG, Eeckman, FH, Kulp, D, Haussler, D, 1997. ``Improved Splice Site Detection in Genie''. *J Comp Biol* **4(3)**, 311-23

Scalzitti, N., Kress, A., Orhand, R. et al. Spliceator: multi-species splice site prediction using convolutional neural networks. BMC Bioinformatics 22, 561 (2021) https://doi.org/10.1186/s12859-021-04471-3

Fairbrother WG, Yeo GW, Yeh R, Goldstein P, Mawson M, Sharp PA, Burge CB. RESCUE-ESE identifies candidate exonic splicing enhancers in vertebrate exons. Nucleic Acids Res. 2004 Jul 1;32(Web Server issue):W187-90. doi: 10.1093/nar/gkh393. PMID: 15215377; PMCID: PMC441531.

**CNF Count Image J Macro**

```
dir = getDirectory("Choose a Directory");
filesArray = getFileList(dir);
for (j=0; j<filesArray.length; j++) {
	setBatchMode(true);
	open(filesArray[j]);
	run("Set Scale...", "distance=471.5 known=500 pixel=1 unit=µm");

	title = getTitle();
	run("Split Channels");
	selectWindow(title + " (green)");
	rename(title+" -muscle fibers");
	run("Enhance Contrast...", "saturated=1 normalize equalize");
	run("Subtract Background...", "rolling=5 sliding");
	run("Gaussian Blur...", "sigma=2");
	run("Smooth");
	wait( 2000 );

	setBatchMode("exit and display");
	run("Morphological Segmentation");
	wait( 2000 );
	call("inra.ijpb.plugins.MorphologicalSegmentation.segment", "tolerance=12.0",
"calculateDams=true", "connectivity=4");
	wait( 2000 );
	//waitForUser("Press okay when segmentation is ready."); //include this pause for
large images, or increase the 'wait' time
	call("inra.ijpb.plugins.MorphologicalSegmentation.setDisplayFormat", "Watershed
lines");
	wait( 2000 );
	call("inra.ijpb.plugins.MorphologicalSegmentation.createResultImage");
	wait( 2000 );
	window = getTitle();
	close("Morphological Segmentation");
	close("Log");
	selectWindow(""+window);
	setBatchMode(true);

	run("Set Scale...", "distance=471.5 known=500 pixel=1 unit=µm");
	run("Find Edges");
	run("Dilate");
	run("Close-");
	run("Invert LUT");
	run("Analyze Particles...", "size=400-15000 circularity=0.4-0.9 show=[Bare Outlines]
exclude clear summarize");
	wait( 2000 );
	run("Duplicate...", " ");
```

```
	run("Invert LUT");
	run("Fill Holes");
	setOption("BlackBackground", true);
	run("Erode");
	wait( 2000 );
	run("Erode");
	wait( 2000 );
	run("Erode");
	wait( 2000 );
	run("Erode");
	wait( 2000 );
	rename("muscle outline");
	wait( 2000 );
	selectWindow(title + " (blue)");
	rename(title+" -total nuclei");
	run("Subtract Background...", "rolling=20");
	run("Gaussian Blur...", "sigma=2");
	run("Smooth");
	run("Find Maxima...", "prominence=10 output=[Maxima Within Tolerance]");
	run("Find Edges");
	run("Close-");
	run("Fill Holes");
	run("Analyze Particles...", "size=5-Infinity show=Masks exclude clear summarize");
	run("Invert LUT");
	rename("Tnuclei");
	imageCalculator("OR create", "muscle outline", "Tnuclei");
	run("Duplicate...", " ");
	run("Fill Holes");
	rename("muscleoutline2");
	imageCalculator("Difference create", "Result of muscle outline","muscleoutline2");
	run("Invert LUT");
	rename(title+" -Cnuclei");
	run("Analyze Particles...", "size=5-Infinity show=Masks exclude clear summarize");
	rename("Cnuclei");
	run("Invert LUT");

	selectWindow("Result of muscle outline");
	run("Set Measurements...", "area standard min feret's redirect=None decimal=3");
	run("Analyze Particles...", "size=400-15000 circularity=0.4-0.9 show=Nothing display
exclude clear include add");
	Table.rename("Results", "OriginalResults");

	CnucleiCount = 0;
	MaxColArray = Table.getColumn("Max");
	for (i = 0; i < MaxColArray.length; i++) {
		if (MaxColArray[i]==255) {
			MaxColArray[i] = 1;
```

```
				CnucleiCount++;
				roiManager("Select", i);
				run("Find Maxima...", "prominence=10 exclude light output=Count");
			} else {
				setResult("Count", i, 0);
				updateResults(); //I think it might be faster to add 0s like this, rather
than allow the find maxima function to do it outside of the if==255 condition
			}
		}
		run("Select None");
		roiManager("reset");
		close("ROI Manager");
		selectWindow("Results");
		NucCountArray = Table.getColumn("Count");
		close("Results");
		Table.rename("OriginalResults", "Results");
		Table.setColumn("CNuclei_Count/fiber", NucCountArray);
		Table.setColumn("Max", MaxColArray);
		Table.sort("CNuclei_Count/fiber");
		Table.renameColumn("Max", "Central Nuclei_Y/N");
		Table.sort("Central Nuclei_Y/N");
		Table.renameColumn("MinFeret", "MinFeret_Diameter(um)");
		Table.renameColumn("Area", "Area(um^2)");
		Table.deleteColumn("Min");
		Table.deleteColumn("StdDev");
		Table.deleteColumn("Feret");
		Table.deleteColumn("FeretX");
		Table.deleteColumn("FeretY");
		Table.deleteColumn("FeretAngle");
		run("Read and Write Excel", "stack_results dataset_label="+title+"
file=[C:/Users/joeya/Desktop/40AKO Muscle test images/Central nuclei
counts/Cnucleidistribution.xlsx]");
		//run("Read and Write Excel", "dataset_label="+title);
		run("Close All");
		close("Results");
		setBatchMode("exit and display");
}
```

**Collagen VI quantification Image J Macro**

```
dir = getDirectory("Choose a Directory");
filesArray = getFileList(dir);
for (j=0; j<filesArray.length; j++) {
	setBatchMode(true);
	open(filesArray[j]);
	run("Set Scale...", "distance=185.6670 known=100 pixel=1 unit=µm");

	title = getTitle();
	run("Split Channels");
	selectWindow(title + " (green)");
	run("Sharpen");
	run("Smooth");
	wait( 2000 );

	setBatchMode("exit and display");
	run("Trainable Weka Segmentation");
	wait( 2000 );
	selectWindow("Trainable Weka Segmentation v3.2.34");
	call("trainableSegmentation.Weka_Segmentation.loadClassifier", 
"C:\\Users\\joeya\\Desktop\\Fiji Macros and Test Images\\ColVI Stained classifier.model");
	wait( 2000 );
	call("trainableSegmentation.Weka_Segmentation.getResult");
	wait( 2000 );
	selectWindow("Classified image");
	run("RGB Color");
	wait( 2000 );
	setBatchMode(true);
	title = getTitle();
	rename("Results1");
	run("Convert to Mask");
	run("Average Thickness");
	run("Measure");
	Table.rename("Results", "OriginalResults");
	selectWindow("Results1-AvgThickness");
	NucCountArray = Table.getColumn("AverageThickness");
	close("Results1-AvgThickness");
	Table.rename("OriginalResults", "Results");
	Table.setColumn("AverageThickness", NucCountArray);
	wait( 4000 );
	Table.renameColumn("%Area", "Stained Area(%)");
	Table.deleteColumn("MinThr");
	Table.deleteColumn("Area");
	Table.deleteColumn("MaxThr");
	wait( 2000 );
```

```
	run("Read and Write Excel", "stack_results dataset_label=Sample_name file=[C:/Users/joeya/Desktop/40AKO Muscle test images/Central nuclei counts/Cnucleidistribution.xlsx]");
	run("Close All");
	close("Results");
	setBatchMode("exit and display");
}
```

**Proteomics analysis**

Each muscle tissue lysate sample was heated at 85 °C with 10 mM tris(2-carboxyethyl) phosphine, then incubated at 23 °C for 30 min with 20 mM iodoacetamide followed by precipitation using the chloroform-methanol method {Wessel, 1984, 6731838}. The precipitate was digested with 2 µg LysC (Fujifilm Wako) in 10 µL 8 M urea and 100 mM 4-(2-hydroxyethyl)-1-piperazineethanesulfonic acid at pH 8.0 for 12 h at 30 °C, then diluted to 80 µL with 100 mM 4-(2-hydroxyethyl)-1-piperazineethanesulfonic acid with 2 µg Tryzean (Sigma) for 8 h at 30 °C and again with 2 µg Tryzean (Sigma) for 8 h at 30 °C. An aliquot of each sample containing 35 ug of peptide, estimated by UV absorbance, was labelled with TMTpro reagents (Thermo Fisher Scientific): four wild type (126 to 128N), four BLAJ (128C to 130N), four KO-1 (130C to 132N) and four KO-2 (132C to 134N). Labelling efficiency greater then 99% was confirmed and the samples were combined and desalted using solid phase extraction (Waters, Sep-Pak C18 200 mg Vac Cartridge). The samples were reconstituted in a solution of 90% acetonitrile, 0.1% trifluoroacetic acid (TFA) for hydrophilic interaction chromatography (HILIC) fractionation.

HILIC fractionation was performed on a Dionex Ultimate 3000 HPLC system with a 250 mm long and 1 mm inside diameter TSKgel Amide-80 column (Tosoh Biosciences). The sample was injected into a 250 µl sample loop. The flow rate was 60 µl/min in Buffer A (90% acetonitrile,

0.1% TFA) for 10 min to load the sample. The gradient was from 100% Buffer A to 60% Buffer A (40% Buffer B, which was 0.1% TFA ion water) for 35 min at a flow rate of 50 µl/min. Fractions were collected into a 96-well plate using a Probot (LC Packings) at 30 s intervals. Peak intensity was monitored by absorbance of UV at 214 nm. The UV signal was used to combine selected fractions into similar amounts of peptide, resulting in eighteen final fractions. Final fractions were dried and reconstituted in 5 µL 0.1% formic acid for LC-MS/MS analysis.

The LC-MS/MS was performed using a Dionex UltiMate 3000 RSLC nano system and Q Exactive Plus hybrid quadrupole-orbitrap mass spectrometer (Thermo Fisher Scientific). An in-house 300 mm long 0.075 mm inside diameter column packed with ReproSil Pur C18 AQ 1.9 µm resin (Dr Maisch, Germany) was used. The column was heated to 50 °C using a column oven (PRSO-V1, Sonation lab solutions, Germany) integrated with the nano flex ion source with an electrospray operating at 2.3 kV. The S lens radio frequency level was 50 and capillary temperature was 250 °C. A 3.5 A 3.5 µL aliquot of each fraction was injected into a 20 µl loop and loaded onto the column in 99% reversed phase buffer A (solution of 0.1% formic acid) and 1% buffer B (solution of 0.1% formic acid, 90% acetonitrile) for 17.5 min at 300 µL/min. The gradient, at 250 µL/min, was from then from 99% buffer A to 94% buffer A in 1 min, to 72% buffer A in 71.5 min, to 65% buffer A in 8 min, to 1% buffer A in 1 min, held at 1% buffer A for 2 min, to 99% buffer A in 1 min and held for 8 min. MS acquisition was performed for the entire 110 min.

Data-dependent MS acquisition was used. The MS scans were at a resolution of 70,000 with an automatic gain control target of 1,000,000 for a maximum ion time of 100 ms from m/z 375 to 1500. The MS/MS scans were at a resolution of 35,000 with an automatic gain control target of 200,000 and maximum ion time of 115 ms. The loop count was 12, the isolation

window was 1.1 m/z, the first mass was fixed at m/z 120 and the normalized collision energy was 30. Singly charged ions and those with charge >8 were excluded and dynamic exclusion was for 35 s.

The raw LC-MS/MS data was processed with MaxQuant v1.6.7.0 using the following settings: variable modifications were oxidation (M), acetyl (protein N-terminus), deamidation (NQ). Carbamidomethyl (C) was a fixed modification. Digestion was set to trypsin/P with a maximum of three missed cleavages. The TMTpro correction factors were entered for lot VB294905. The minimum reporter peptide ion faction was 0.6. The *Mus musculus* reference proteome with canonical and isoform sequences downloaded Dec 26 2020 was used. The inbuilt contaminants fasta file was used. The minimum peptide length was 6 and maximum peptide mass was 6000 Da. Second peptides search and dependent peptides searches were enabled. Peptide spectrum matching and protein false discovery rates were set at 1%. All modified peptides and counterpart non-modified peptides were excluded from protein quantification. All other settings were default. The MaxQuant proteinGroups.txt file

entries were filtered: CON_ and REV_ entries were removed. Entries with no intensity value were removed. Corrected reporter ion intensities were used as input for an R script that used limma statistics to statistically compare protein relative intensity between the mice and has been reported previously (https://www.biorxiv.org/content/10.1101/2021.05.16.444356v1).

**RUV bioinformatics analysis of proteomics raw data**

The Remove Unwanted Variation (RUVIII) method (1) from the ruv R package (2) was used to remove batch-batch experimental variations. This method relies on a set of proteins with minimal change in abundance between different cell types or experimental treatments, which are called negative control genes, to measure the background level of unwanted variations.

An initial analysis of variance (ANOVA) analysis comparing all experimental groups was used to identify the negative control proteins, which were the bottom 300 ranked proteins which had the least statistically significant q-values from the ANOVA test. Three unwanted factors from the first experiment and five unwanted factors were removed from the first and second replicate datasets, respectively. The resulting RUVIII adjusted abundance matrix was used to perform differential protein abundance analysis and all subsequent analyses. Differential protein abundance analyses were performed by comparing a pair of sample groups using a linear model fitted using the 'lmFit' function from the 'limma' R/Bioconductor package (3). The protein-centric p-values were calculated using the empirical Bayes method and multiple testing correction was applied using the q-values (4).

**References**


1. Molania, R., et al., *A new normalization for Nanostring nCounter gene expression data.* Nucleic Acids Res, 2019. **47**(12): p. 6073-6083 DOI: 10.1093/nar/gkz433.
2. Gagnon-Bartsch, J.A. and T.P. Speed, *Using control genes to correct for unwanted variation in microarray data.* Biostatistics, 2012. **13**(3): p. 539-52 DOI: 10.1093/biostatistics/kxr034.
3. Ritchie, M.E., et al., *limma powers differential expression analyses for RNA-sequencing and microarray studies.* Nucleic Acids Research, 2015. **43**(7): p. e47-e47 DOI: 10.1093/nar/gkv007.
4. Storey, J.D., *A direct approach to false discovery rates.* Journal of the Royal Statistical Society: Series B (Statistical Methodology), 2002. **64**(3): p. 479-498 DOI: https://doi.org/10.1111/1467-9868.00346.
