# Supplementary material for: Minimal expression of dysferlin prevents development of dysferlinopathy in dysferlin exon 40a knockout mice
Source: Acta Neuropathol Commun. 2023 Jan 18;11:15. doi: 10.1186/s40478-022-01473-x (PMC9847081; doi:10.1186/s40478-022-01473-x)
Supplement: Supplementary file 8 — Additional file 8. Supplementary Table 1. PCR primers used to probe dysferlin Exon 40a locus. [file 40478_2022_1473_MOESM8_ESM.pdf]

Supplementary Table 1- PCR primers used to probe dysferlin Exon 40a locus

Name	PCR/RT-PCR	Sequence 5’-3’
Guide #2 F	PCR	TAAACATGCCGGTTAGCAAC
Guide #9 F	PCR	ACAGATGGGCTGTCAAGCTT
Guide common R	PCR	AAAAGCACCGACTCGGTGCC
Dysf 8F	PCR	GGAGAACCCTACTCCCTCA
Dysf 3R	PCR	CCTCTCCTCCCTACCCTTTG
Dysf ex38F	RT-PCR	GTGCAGTCCTGTGTCATCAGAAAC
Dysf ex39F	RT-PCR	CAGTTGTGGGTCAGTGTACCATTC
Dysf ex42R	RT-PCR	TGGATCATCTGTCTCCTCCTGA
Dysf ex43R	RT-PCR	AGCCAAATGCTCGGACGATATAA
Dysf 40aF	RT-PCR	GGAGAACTTCCTGTGCGACC
Dysf 40aR	RT-PCR	CTTTTCCCTCTCCCCGACAGAAGCAAAGAA
