# Supplementary material for: Minimal expression of dysferlin prevents development of dysferlinopathy in dysferlin exon 40a knockout mice
Source: Acta Neuropathol Commun. 2023 Jan 18;11:15. doi: 10.1186/s40478-022-01473-x (PMC9847081; doi:10.1186/s40478-022-01473-x)
Supplement: Supplementary file 15 — Additional file 15. Copy of unedited blots and gels. [file 40478_2022_1473_MOESM15_ESM.pdf]

Full unedited gel for Supplementary Figure 1B (left panel)

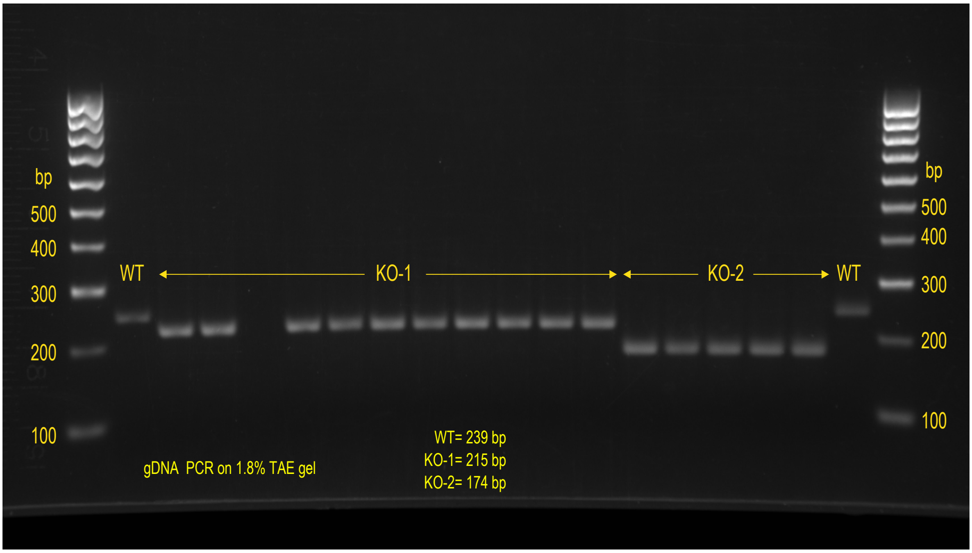

Full unedited gel for Supplementary Figure 1B (right panel)

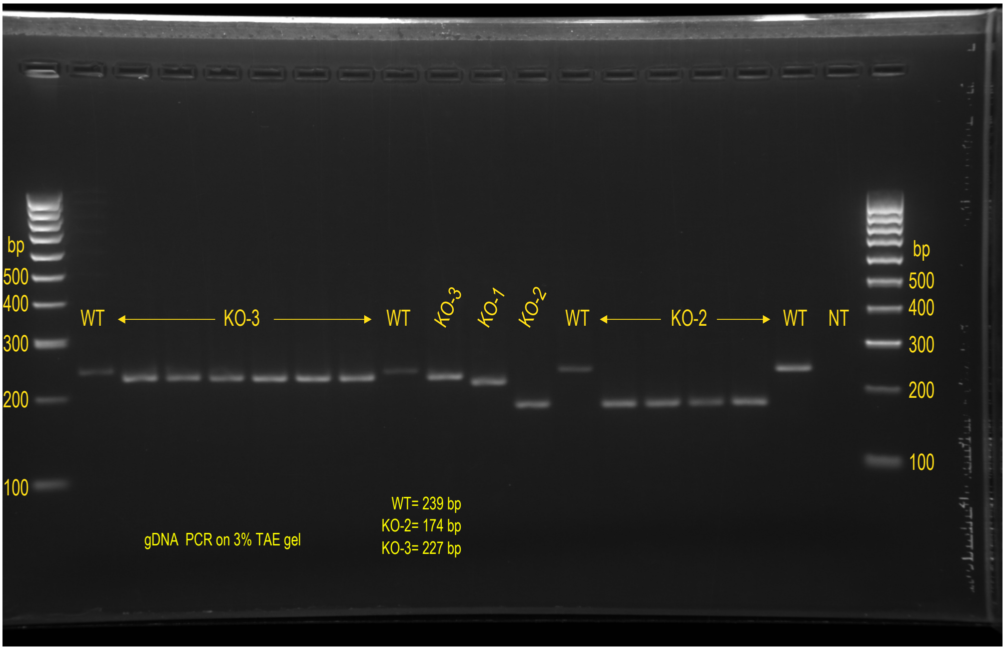

Full unedited gel for Supplementary Figure 1C

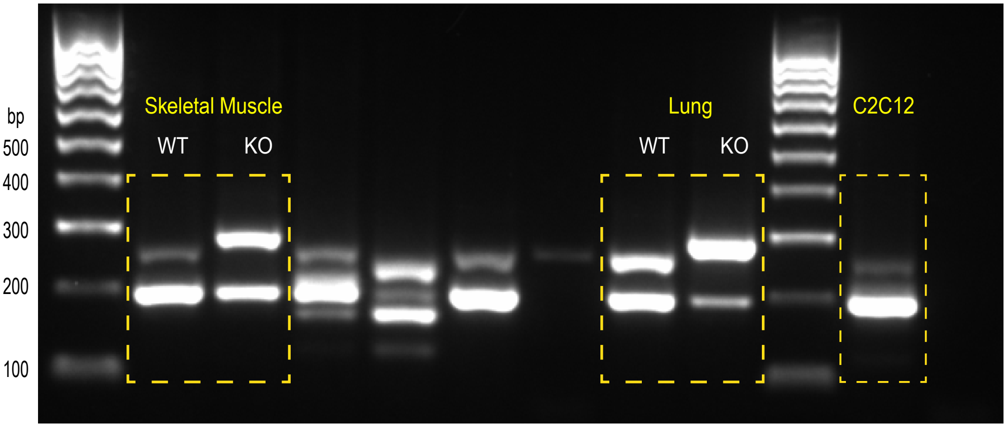

Lanes represented in Supplementary Figure 1C are highligted by yellow bound box

Full unedited gel for Supplementary Figure 1D

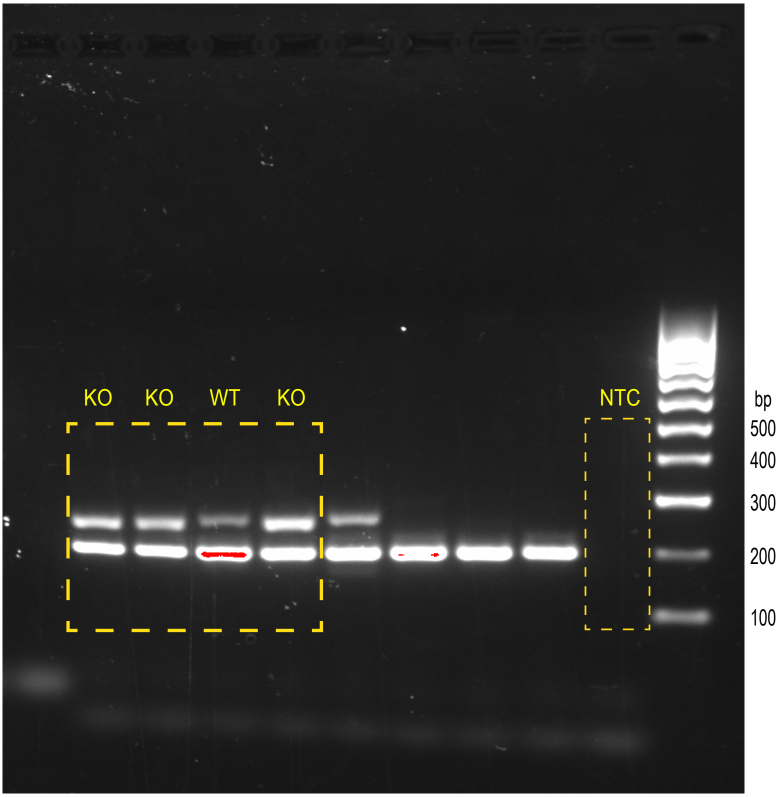

Lanes represented in Supplementary Figure 1D are highligted by yellow bound box

Full unedited gel for Supplementary Figure 1E

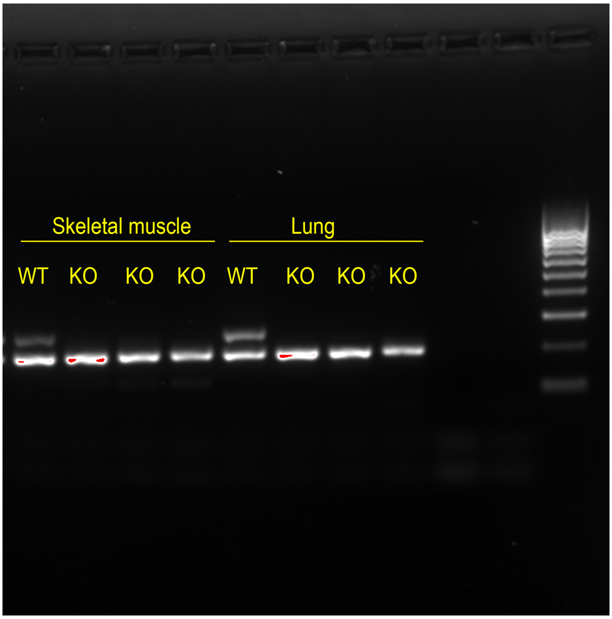

Full unedited blot for Supplementary Figure 2B Quadriceps panel

Probed with Hamlet-1 (anti-dysferlin)

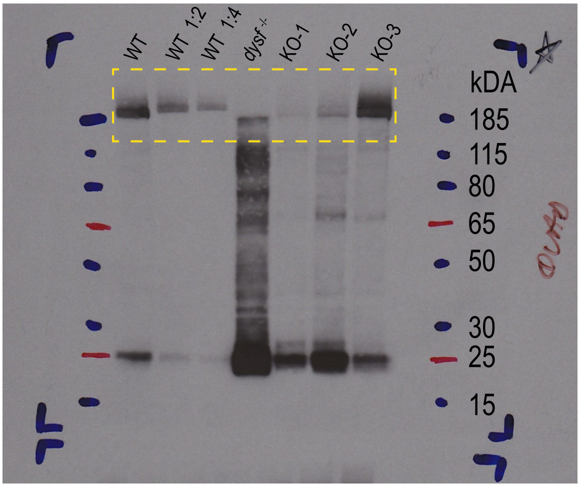

Probed with Beta Actin loading control

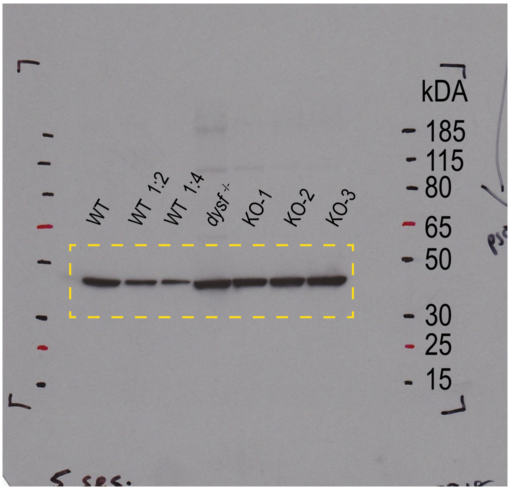

Lanes represented in Supplementary Figure 2B are highligted by yellow bound box

Full unedited blot for Supplementary Figure 2B Psoas panel

Probed with Hamlet-1 (anti-dysferlin)

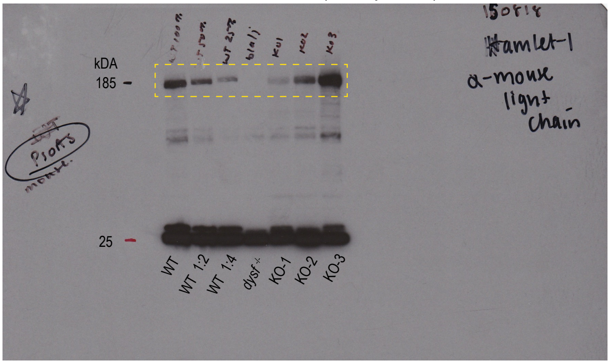

Probed with Beta Actin loading control

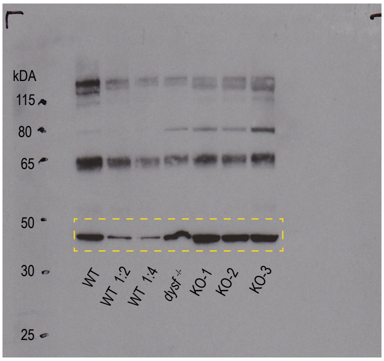

Lanes represented in Supplementary Figure 2B are highligted by yellow bound box

Full unedited blot for Supplementary Figure 2B Heart and Spinalis panel

Probed with Hamlet-1 (anti-dysferlin)

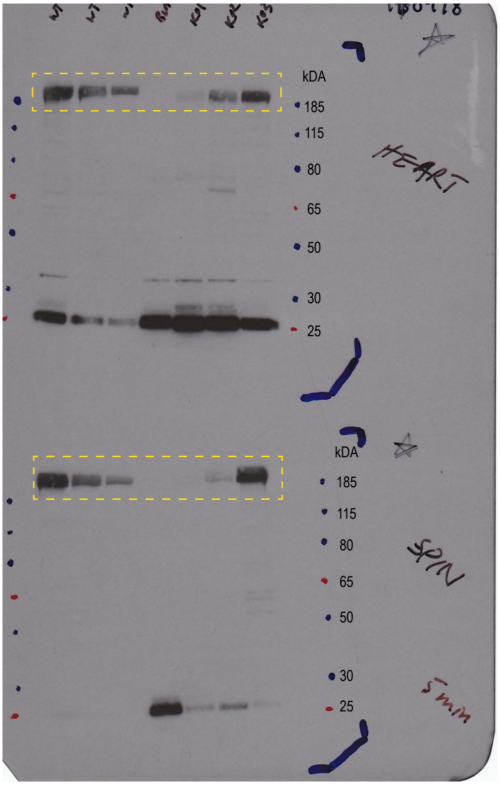

Probed with Beta Actin loading control

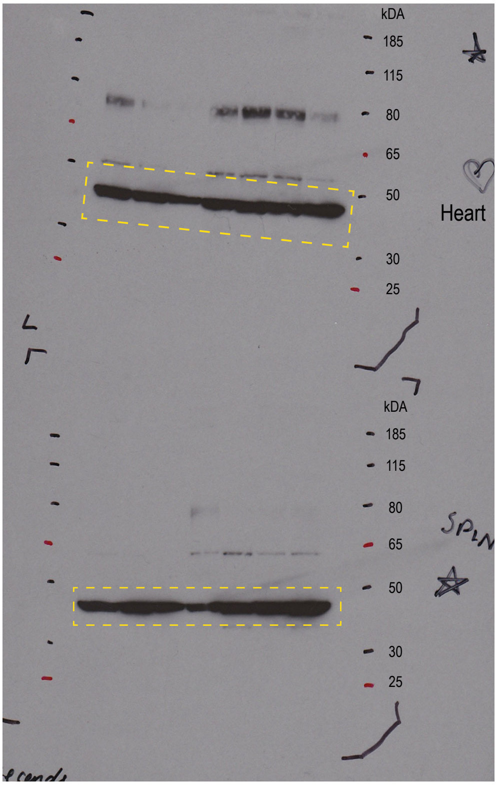

Lanes represented in Supplementary Figure 2B are highligted by yellow bound box
